# Identification and analysis of the stigma and embryo sac-preferential/specific genes in rice pistils

**DOI:** 10.1186/s12870-017-1004-8

**Published:** 2017-03-07

**Authors:** Li Yu, Tengfei Ma, Yuqin Zhang, Ying Hu, Ke Yu, Yueyue Chen, Haoli Ma, Jie Zhao

**Affiliations:** State Key Laboratory of Hybrid Rice, College of Life Sciences, Wuhan University, Wuhan, 430072 China

**Keywords:** Embryo sac, female gametophyte, novel gene, rice, RNA-Seq, Stigma

## Abstract

**Background:**

In rice, the pistil is the female reproductive organ, and it consists of two stigmas and an ovary. The stigma is capable of receiving pollen grains and guiding pollen tube growth. The ovary holds the embryo sac, which is fertilized with male gametes to produce seed. However, little is known about the gene function and regulatory networks during these processes in rice.

**Results:**

Here, using the RNA-Seq technique, we identified 3531 stigma-preferential genes and 703 stigma-specific genes within the rice pistils, and we verified 13 stigma-specific genes via qRT-PCR and *in situ* hybridization. The GO analysis showed that the transport-, localization-, membrane-, communication-, and pollination-related genes were significantly enriched in the stigma. Additionally, to identify the embryo sac-preferential/specific genes within the pistils, we compared a wild-type ovary with a mutant *dst* (*defective stigma*) ovary and found that 385 genes were down-regulated in *dst*. Among these genes, 122 exhibited an ovary-specific expression pattern and are thought to be embryo sac-preferential/specific genes within the pistils. Most of them were preferentially expressed, while 14 of them were specifically expressed in the pistil. Moreover, the rice homologs of some *Arabidopsis* embryo sac-specific genes, which played essential roles during sexual reproduction, were down-regulated in *dst*. Additionally, we identified 102 novel protein-coding genes, and 6 of them exhibited differences between the stigma and ovary in rice as determined using RT-PCR.

**Conclusions:**

According to these rice ovary comparisons, numerous genes were preferentially or specifically expressed in the stigma, suggesting that they were involved in stigma development or pollination. The GO analysis indicated that a dry rice stigma might primarily perform its function through the cell membrane, which was different from the wet stigma of other species. Moreover, many embryo sac-preferential/specific genes within the pistils were identified and may be expressed in female rice gametophytes, implying that these genes might participate in the process of female gametophyte specialization and fertilization. Therefore, we provide the gene information for investigating the gene function and regulatory networks during female gametophyte development and fertilization. In addition, these novel genes are valuable for the supplementation and perfection of the existing transcriptome in rice, which provides an effective method of detecting novel rice genes.

**Electronic supplementary material:**

The online version of this article (doi:10.1186/s12870-017-1004-8) contains supplementary material, which is available to authorized users.

## Background

In angiosperms, the life cycle consists of a haploid gametophyte stage and a diploid sporophyte stage. The male gametophyte (pollen grain) is composed of two sperm cells and one vegetative cell, whereas the female gametophyte (mature embryo sac) is composed of one egg cell, two synergid cells, one central cell with two nuclei, and three or more antipodal cells [1–4]. After the maturation of the gametophytes, pollen grains are transferred into the stigma, and then they emit pollen tubes and grow into the transmitting tissue. After that, one pollen tube grows into one of the synergid cells and releases two sperm cells. Ultimately, one sperm cell fuses with the egg cell to form the zygote, while the other fuses with the central cell to form the primary endosperm nucleus; these fusions then lead to the production of the embryo and endosperm, respectively [5]. During this process, communication between the pollen grain and pistil occur closely and frequently to maximize reproductive success.

The pollen grain-stigma interaction occurs in the stigma, which includes two types, namely the wet stigma (e.g., in *Nicotiana tabacum* and *Lilium longiflorum*) and the dry stigma (e.g., in *Arabidopsis thaliana*, *Zea mays* and *Oryza sativa*). When pollen grains are deposited on papilla cells, many stigma-preferential/-specific genes begin to be expressed and play vital roles. The abnormal expression or absence of these genes or secondary metabolites would disrupt the development or function maintenance of the stigmas. In *Brassica*, a receptor-like kinase (RLK) called the S-receptor kinase (SRK) binds the ligand, SCR/SP11, to phosphorylate ARC1 (armadillo repeat-containing protein1), and then ARC1 mediates the degradation of the exocyst subunit genes, such as Exo70A1, which results in the recognized failure of compatible pollen grains [6–11]. Similarly, the other two stigma-specific glycoproteins, SLG (S-locus glycoprotein) and SLRI (S-locus-related 1 protein), disturb the adhesion of incompatible pollen grains via interactions with pollen coat proteins in *Brassica* [12–15]. In tobacco and petunias, STIG1 (stigma-specific gene 1) is involved in the regulation of pistil exudates to affect the growth of pollen tubes [16, 17]. Additionally, many secondary metabolites, such as sulfinylated azadecalin, are also essential for the germination and growth of pollen grains [18].

After the pollen grains germinate, the pollen tubes grow into the transmitting tissues toward the embryo sac. Signal molecules from the pistil function in prerequisite roles for pollen tube growth, especially the guidance and termination of the pollen tubes [19–21]. Peptides and glycoproteins are star molecules, which are necessary for the growth and guidance of pollen tubes [22]. In *Arabidopsis*, CLV3/ESR-related peptide (CLE45) and vacuolar protein sorting 41 (AtVPS41) promote the growth of pollen tubes, while pollen tubes germinate and grow normally in *ccg*, *myb98* and *feronia*, but they cannot reach the synergid cell in the first two mutants and overgrow after arriving at the synergid cell in *feronia* [23–27]. In *Nicotiana*, arabinogalactan proteins called TTS are involved in the growth of pollen tubes and may function as a Ca^2+^ flux capacitor in the extracellular matrix [28–30]. In *Torenia fournieri*, *Torenia concolor*, and *Brassica*, the LURE peptides from synergid cells are similar defensin-like CRPs, and they play an essential role in pollen tube attraction [31–33]. In monocots, ZmES4 induces rapid pollen tube rupture in maize, and OsPTB1 (POLLEN TUBE BLOCKED 1) promotes pollen tube growth to control the rice seed-setting rate [34, 35].

Rice is a model monocotyledon and one of the most important crops. *OsIG1*, *OsMADS13*, and *OsLOG* play essential roles during the biogenesis of the rice ovule [36–39]. To date, approximately 30 genes have been found to participate in the pollen meiosis process, and they are likely involved in the development of female gametophytes in rice [40, 41]. However, all of them are involved in the process before the cell differentiation of the female gametophyte. Although the killer-protector system at the rice S5 locus and OsDEES1 has provided insights into embryo sac development, the mechanism of cell differentiation and the unique features of different cells in the embryo sac are still unclear [42–44]. Additionally, few researchers have reported on the development mechanism of the stigmas.

In *Arabidopsis*, a series of female gametophyte-specific genes were identified via a comparison of the ovules with the embryo sac and without it, with the aim of dissecting the genes and the regulatory networks of certain biologic processes. Of these genes, three transcription factors, namely, MYB98, MYB64, and AGL61, have been confirmed to play essential roles in the development of female gametophytes [23, 45–47]. Similarly, in maize, the egg cell-specific gene ZmEA1 and the synergid-specific gene ZmES4 are required for completing fertilization [34, 48]. In rice using microarrays and RNA sequencing (RNA-Seq), only two female gametophyte-specific genes, *ECAGL1* (LOC_Os03g18530) and a SCP-like gene (LOC_Os04g22220), in addition to some stigma-preferential genes, were identified [49–51]. However, to date, no stigma-specific gene has been reported.

In this study, to identify rice genes that are specifically expressed in the stigma and embryo sac, we adopted RNA-Seq and qPCR techniques to analyze the gene expression in the pistils, ovaries, and stigmas of the rice variety “Hwayoung” and in ovaries from the *dst* mutant. We obtained a series of stigma-preferential or -specific genes within the rice pistils and verified their expression via qRT-PCR and *in situ* hybridization. Furthermore, the GO analysis showed that the transport-, localization-, membrane-, communication-, and pollination-related genes were significantly enriched in the stigma. In addition, we found that many ovary-specific genes were down-regulated in the mutant *dst* compared to the HY ovary, and we believed that they were embryo sac-preferential/specific genes within the pistils. Most of them were preferentially/specifically expressed in the pistil, implying that they play an essential role during female gametophyte development and fertilization in rice. Additionally, we identified many novel protein-coding genes and verified them by using RT-PCR, displaying their value in the supplementation and perfection of the existing transcriptome in rice and providing an effective method to detect novel rice genes.

## Results

### Strategy to identify stigma-specific and embryo sac-preferential/specific genes within the pistils

**Fig. 1:**
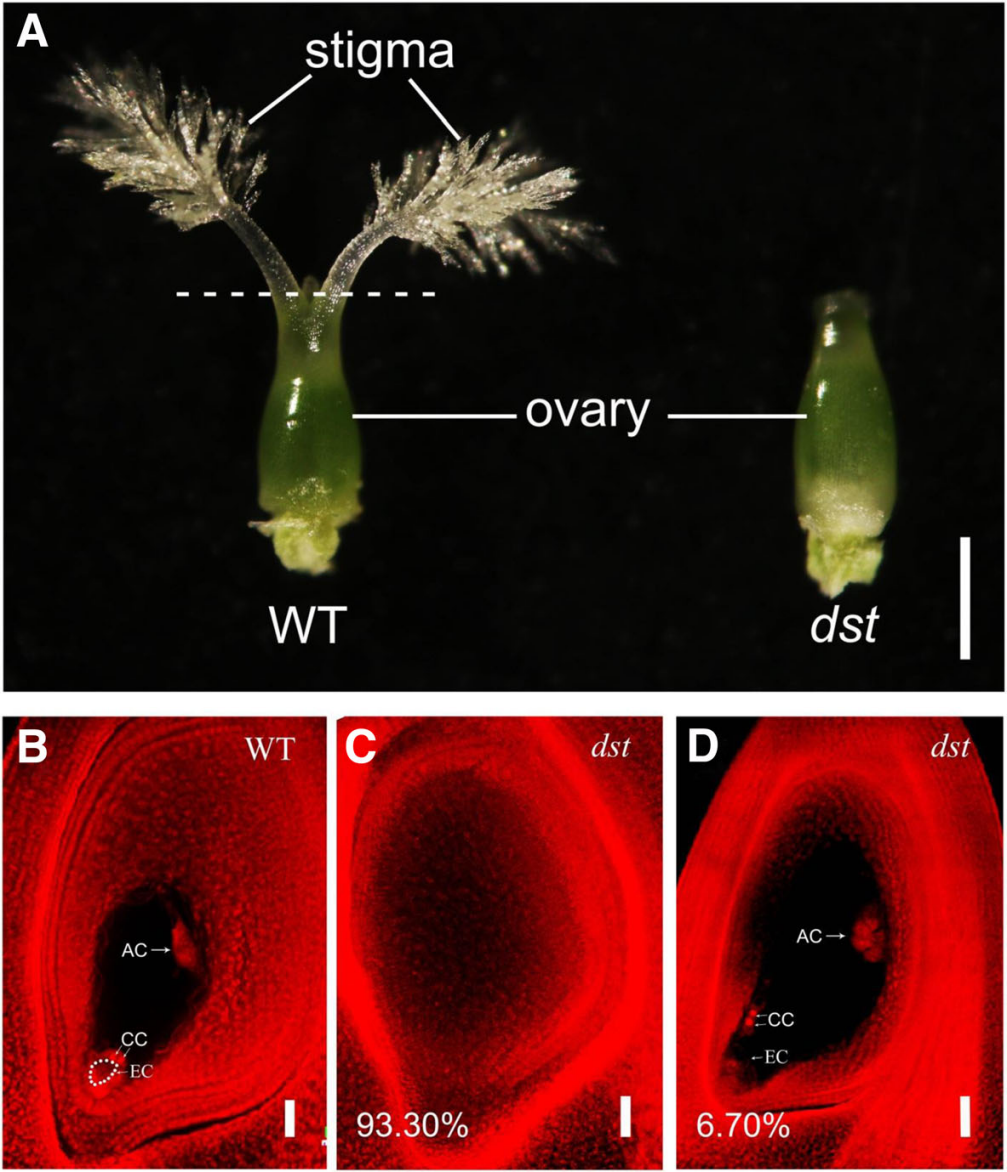
Phenotype of *dst* mutant. **a**, Phenotypes of pistils in wild type and mutant *dst* (without stigma). **b**, Embryo sac of wild type. **c** and **d**, Embryo sacs of mutant *dst*. WT, wild type; AC, antipodal cell; CC, central cell; EC, egg cell. Bar was 500 μm in a, 20 μm in b-c and 50 μm in **d**

In our study, a rice mutant without a stigma was obtained and named *dst* (*defective stigma*) (Fig. 1a). Scanning confocal microscopy analysis revealed that 93.6% of the ovules in the *dst* (*n* = 179) lacked embryo sacs (, while 6.7% of the ovules in the *dst* presented embryo sacs (Fig. 1b-d). To obtain the embryo sac-specific genes within the pistils, we identified genes that were specifically expressed in the ovary and down-regulated in the ovary without an embryo sac compared to the wild type ovary. The approach to obtaining the stigma-specific genes within the pistils was to identify the mRNAs that were present in the stigma but not in the ovary.

**Fig. 2:**
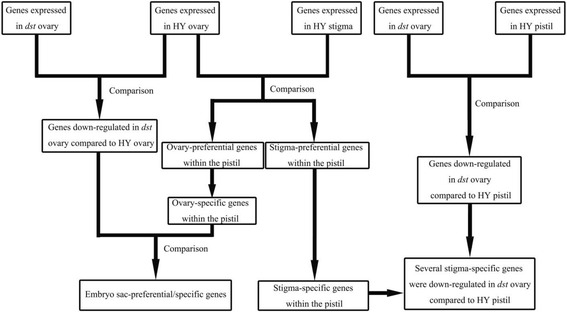
Strategy to identify embryo sac-preferential/specific and stigma-specific genes

First, using RNA-Seq technology, we obtained gene expression data from the following four samples: a Hwayoung (HY) pistil, HY stigma, HY ovary, and *defective stigma* (*dst*) ovary (without stigma). Then, three classes of comparisons were performed as follows: HY ovary vs. HY stigma, HY pistil vs. *dst* ovary, and HY ovary vs. *dst* ovary. From the first class, ovary-/stigma-preferential and ovary-/stigma-specific genes within the pistils were obtained. To ensure that the expression of the stigma-specific genes was dependent on the stigma, we performed a second comparison, which showed that many stigma-specific genes were down-regulated in the *dst* ovary compared to the HY pistil. Additionally, with the help of the third class, we identified the genes that were down-regulated in the *dst* ovary compared with the HY ovary. Simultaneously, some ovary-specific genes were also down-regulated in the *dst* ovary compared to the HY ovary, where the embryo sac-preferential/specific genes are believed to be within the pistil (Fig. 2).

Based on previous studies, we found that *ECAGL1* (LOC_Os03g18530) is specifically expressed in the egg cell, an SCP-like gene (LOC_Os04g22220) is a synergy cell-specific gene, and *OsMADS13* (LOC_Os12g10540) is preferentially expressed in the ovule [36, 50]. In our study, *ECAGL1* and LOC_Os04g22220 exhibited ovary-specific expression patterns, and their expressions in the HY ovary were found to be more than 2 times that in the *dst* ovary (Additional file 1: Table S1). However, OsMADS13 was abundantly expressed in the HY and *dst* ovaries with no significant difference (Additional file 1: Table S1). This finding provided a validation of our strategies and indicated that these embryo sac-preferential/specific genes may exhibit embryo sac-specific expression patterns.

### Global analysis of RNA-Seq data

To obtain the RNA-Seq data, we prepared the following 4 tissues: HY pistil (Pi), HY stigma (St), HY ovary (O), and *dst* ovary (*dst*) tissues. As shown in Fig. 1a, the HY pistil was cut at the dotted line to divide it into two samples; the upper part was classified as the stigma, and the lower part was used as the ovary. Two independent cDNA libraries for each sample were built, and more than 53 million reads of 125 bp each were obtained for every library. Among them, 89.35% ~ 90.22% of the high quality reads were mapped onto the rice genome (http://rice.plantbiology.msu.edu/pub/data/Eukaryotic_Projects/o_sativa/annotation_dbs/pseudomolecules/version_7.0/all.dir/), and 85.63% ~ 86.43% were uniquely mapped onto the rice genome (Table 1). The rice transcriptome that was downloaded from the RGAP (Rice Genome Annotation Project) website (http://rice.plantbiology.msu.edu/pub/data/Eukaryotic_Projects/o_sativa/annotation_dbs/pseudomolecules/version_7.0/all.dir/) was used as the reference transcriptome [52].

**Table 1:** Mapping the results of the RNA-Seq reads

Samples	Pi-1	Pi-2	*dst −*1	*dst −*2	O-1	O-2	St-1	St-2
Total reads	58774736	61359244	53863524	62151522	57441280	62261406	59206314	69461036
Total mapped	53025124 (90.22%)	55203250 (89.97%)	48463393 (89.97%)	55804589 (89.79%)	51325197 (89.35%)	56054854 (90.03%)	53334672 (90.08%)	62416104 (89.86%)
Uniquely mapped	50797925 (86.43%)	52875834 (86.17%)	46444486 (86.23%)	53514080 (86.10%)	49188046 (85.63%)	53491494 (85.91%)	50859896 (85.90%)	59648188 (85.87%)
Multiple mapped	2227199 (3.79%)	2327416 (3.79%)	2018907 (3.75%)	2290509 (3.69%)	2137151 (3.72%)	2563360 (4.12%)	2474776 (4.18%)	2767916 (3.98%)

To assess the gene expression in different tissues, the FPKM (fragments per kilobase of gene per million) of each gene were calculated as the normalized read counts. The total length of the exon region was considered to be the length of the gene, and genes with FPKM values lower than 0.5 were not considered to be expressed genes [53]. To evaluate the threshold, 5 genes with no expression in the rice pistil or panicle were examined [54–56]. Most genes were not detected in the 6 HY libraries, and the highest expression was 0.37 (Additional file 2: Table S2). All of the above results suggest that the threshold is useful for distinguishing among genes that are with or without expression.

**Fig. 3:**
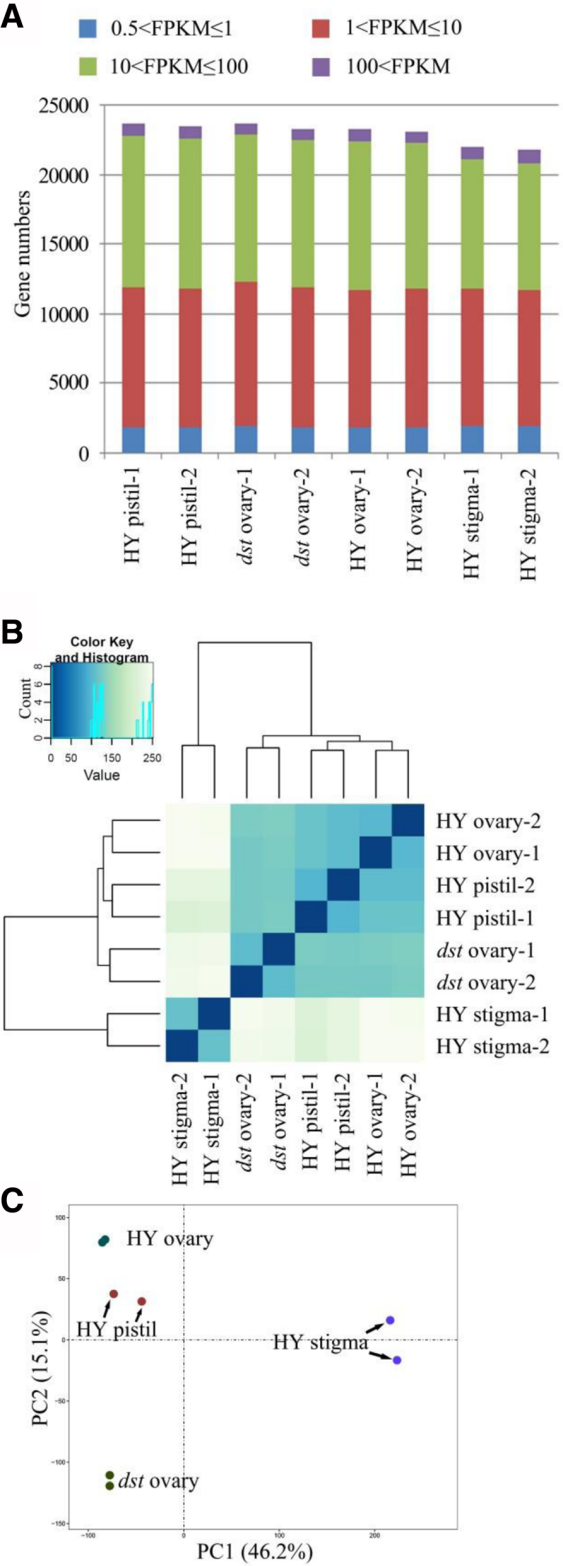
Global analysis of transcriptome. **a**, The number of expressed genes and respective expression levels in each sample based on the FPKM. **b**, The hierarchical cluster analysis of samples. **c**, The principal component analysis. The first and second principal components explained 46.2% and 15.1% of variance, respectively. PC1, the first principal component; PC2, the second principal component

Using the 0.5 FPKM as the threshold, more than 23,000 genes were found to be expressed in the HY pistil, *dst* ovary, HY ovary, and HY stigma. The expression distribution was similar among the six samples in the pistil and ovary, but the fewest genes were expressed in the stigma with a significantly reduced number of genes whose FPKM values were approximately 10 ~ 100, indicating that there were different physiological activities in the stigma (Fig. 3a). A saturation analysis was used to evaluate whether the reads were sufficient to calculate the expression of the genes correctly [57], and all of the transcripts with different expressions showed low relative error when the resampling percentage was more than 90%, even if the gene expression was very low (Additional file 3: Figure S1). To assess the overall relations of the transcriptomes from different tissues, a hierarchical cluster analysis of the samples was performed (Fig. 3b). Two biological replicates of every tissue were clustered together. The HY stigma was located on an independent branch, whereas the other samples were located on another branch, which was divided into two smaller branches. One branch was the *dst* ovary, and the other was the HY pistil and HY ovary. The principal component analysis (PCA) showed a similar result. The HY stigma was located around the junction of the first and the fourth quadrants, the *dst* ovary was located in the third quadrant, the HY ovary was located at the top part of the second quadrant, and the HY pistil was located in the middle and lower part of the second quadrant. All of the results indicated that there was good repeatability among the biological replicates and differences between the tissues (Fig. 3c).

### Identification and analysis of stigma-/ovary-preferential genes within the pistils

To find the genes that are preferentially expressed in the ovary and stigma, we compared the HY ovary and HY stigma. The results showed that 3793 and 3531 genes were highly expressed in the ovary and the stigma, respectively (Additional file 4: Figure S2A and Additional file 5: Table S3 and Additional file 6: Table S4). To obtain further insight regarding the functional differences in tissue-preferential genes, the WEGO (Web Gene Ontology Annotation Plot) online tool was used to classify the overrepresented GO (Gene Ontology) terms [58].

**Fig. 4:**
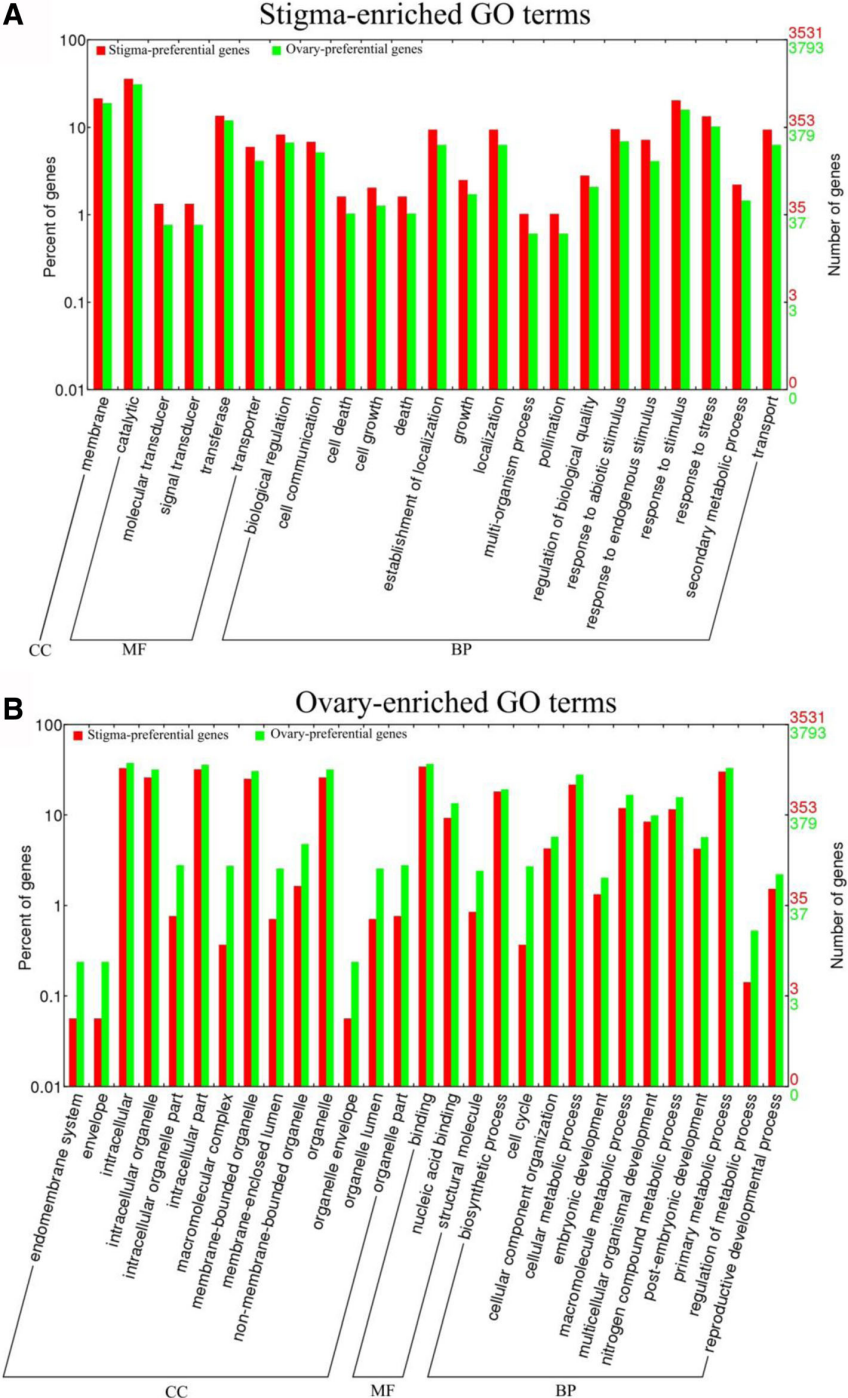
Gene ontology (GO) enrichment analysis of stigma-preferential and ovary-preferential genes. **a**, The stigma-enriched GO terms. **b**, The ovary-enriched GO terms. The results are summarized in three main categories: cellular component (CC), molecular function (MF) and biological process (BP). The y-axis indicates the number and percentage of genes in a category. Comparison of GO function categories between ovary- and stigma-preferential genes was calculated using the WEGO online webtool based on Pearson Chi-Square test. The GO terms were removed if any of the expected counts were less than 5 or the p-value was more than the significant level of 0.05

Based on the cellular component (CC), only one GO term was enriched in the stigma (“membrane”), whereas 14 GO terms of the cellular component were overrepresented in the ovary (Fig. 4), suggesting that the functions of the stigma were primarily executed through the membrane. Based on the molecular function (MF), there were five GO terms that were enriched in the stigma as follows: “catalytic”, “molecular transducer”, “transferase”, “signal transducer”, and “transporter” (Fig. 4a). This finding suggested the existence of vigorous metabolic activities in the stigma, in which small molecule metabolites, such as secreted peptides, were synthesized and then transported to particular locations in the extracellular region or membrane. During the process, transporters played essential roles while there were 210 transporter-related genes were enriched in stigma (Additional file 6: Table S4). By contrast, the three ovary-enriched terms were divided into two parts, in which two were related to the binding activity and the other one was related to structural molecules (Fig. 4b).

Based on the biological process (BP), seventeen GO terms were overrepresented in the stigma. Among them, there were two GO terms associated with the location, which implied that the gradient or polarity distribution was widespread in the stigma and was necessary for sexual reproduction. Additionally, “pollination”, the four terms of response to stimulus or stress, “cell communication”, and “secondary metabolic process” were all overrepresented in the stigma. The former two terms implied that the function of the stigma was to respond to a stimulus (including pollen grains) and recognize compatible pollen grains. The latter two terms implied that communication and secondary metabolites may play essential roles in the stigma-development and pollination. Some evidence was found in other species. When pollen grains adhered to the stigma, communication developed, and recognition factors, such as the SLG and SLR1 proteins in *Brassica*, distinguished among the compatible and incompatible pollen grains; however, the nature of the recognition system was not clear [12, 14]. In addition, the stigma with abnormal exudates also affected the pollination, as in the STIG1 knockdown or knockout plant in tobacco and petunias [16, 17]. All of the above results implied that secretion might perform an important function in the stigma, while many genes, such as Rab GTPases, SNARE complexes and exocyst subunits, played essential roles in secretion and maintaining the normal function of the stigma [11, 59]. However, there was no GO annotation of secretion in rice, and thus we downloaded all the *Arabidopsis* protein sequences under the GO term “secretion” (GO: 0046903), used the BLASTP tool with an E-value of 0.00001, and then found 647 genes that were related to secretion (Additional file **7**: Table S5). Among these genes, there were 40, 28 and 55 genes coding proteins homologous to Rab GTPases, SNARE complexes and exocyst subunits, respectively [11, 59, 60]. Interestingly, the secretion-related genes, including Rab GTPases, SNARE complexes and exocyst subunits, were overrepresented in the stigma (Additional file **7**: Table S5). By contrast, the GO terms related to the biosynthetic process, metabolic process, cellular component synthesis, and organization were enriched in the ovary, implying that they played roles in the ovary and embryo sac (Fig. 4b).

Moreover, the analysis of the KEGG (Kyoto Encyclopedia of Genes and Genomes) pathways provided a confirmation of the GO analysis. The stigma-enriched pathways were primarily associated with basal and secondary metabolism, which may synthesize nutrients and signal molecules that functioned in the recognition system or guidance system of pollen grains, and the pathways were also associated with the “phagosome” and “plant hormone signal transduction” (Additional file 8: Table S6). By contrast, the enriched pathways in the ovary were primarily divided into two categories. One category was photosynthesis and the other was the cell cycle, and the cell cycle was separated into three classes (DNA metabolism, RNA metabolism, and protein synthesis) (Additional file 9: Table S7).

### Identification of stigma-/ovary-specific genes within the pistil

To determine the stigma-specific genes within the pistil, we selected stigma-preferential genes, the expression of which in any replicate of the ovary was lower than 0.5 and more than 0.5 in any replicate of the stigma, and we identified 703 stigma-specific genes (Additional file 10: Table S8). Using a similar method, 1257 ovary-specific genes were identified (Additional file 11: Table S9). Based on the biological process, the stigma-specific genes were enriched in 18 GO terms, and the top two results included the “metabolic process” and “pollination”, whereas the ovary-specific genes were overrepresented in 24 GO terms. The top two findings included the “DNA metabolic process” and the “cell cycle” (Additional file 12: Figure S3A-B).

**Fig. 5:**
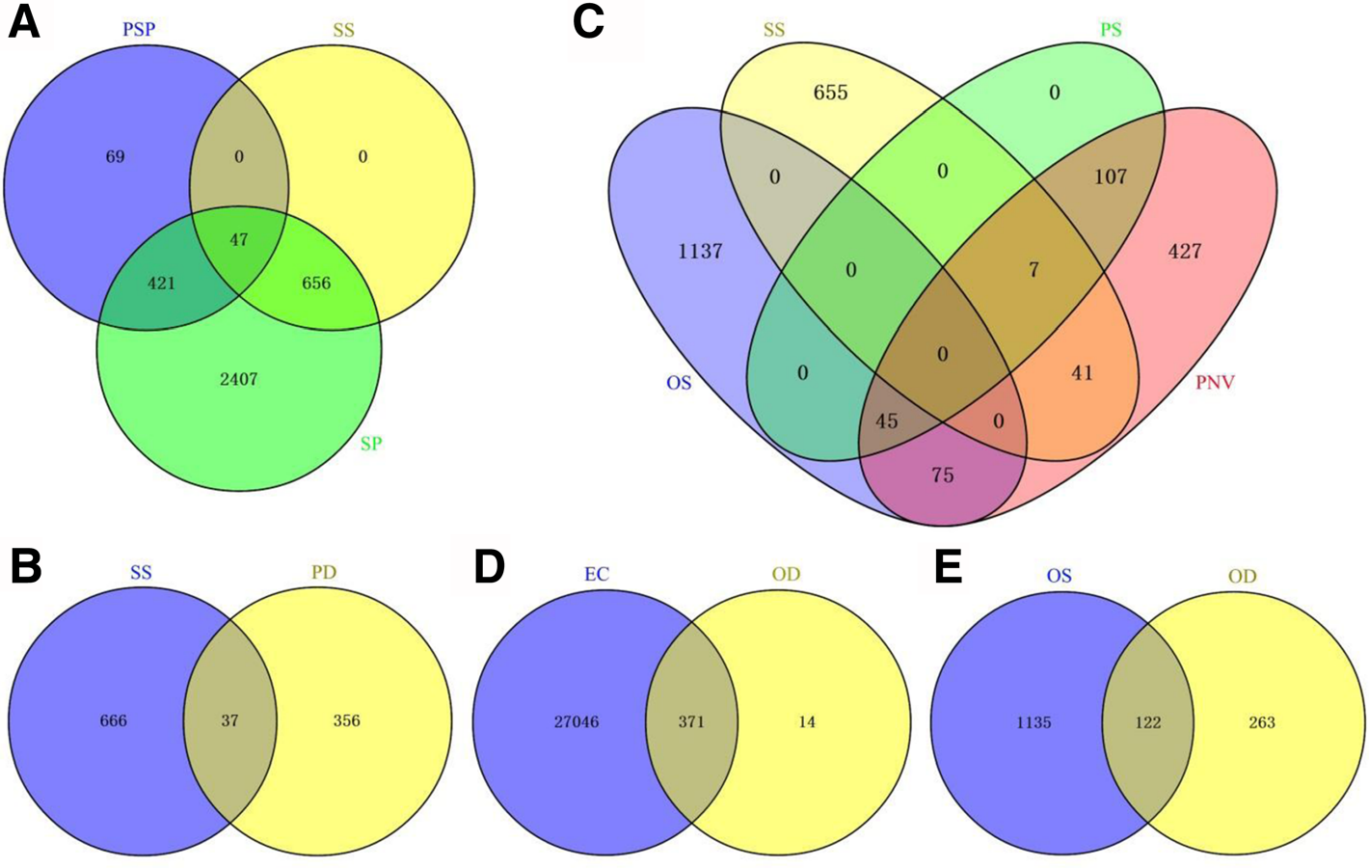
Venny analysis. **a**, There were 537 plumose stigma-preferential genes in rice [49]. Here, 421 from 3531 stigma-preferential genes (*green ring*) and 47 from 703 stigma-specific genes (*yellow ring*) within rice pistils belonged to plumose stigma-preferential genes. **b**, Among the 703 stigma-specific genes (*blue ring*), 37 genes were down-regulated in *dst* compared to HY pistil. **c**, Among these pistil-specific genes, there were 45 genes with ovary-specific expression and 7 genes with stigma-specific expression. Besides, there were 75 ovary-specific genes and 41 stigma-specific genes in pistil-expressed genes with no expression in vegetative tissues. **d**, Among the 385 genes (*yellow ring*) down-regulated in *dst* compared to HY ovary, 371 genes were present in egg cell. **e**, Among the 385 genes (*yellow ring*) down-regulated in *dst* compared to HY ovary, 122 genes were specially expressed in ovary. EC, genes expressed in the egg cell; OD, genes down-regulated in *dst* compared to HY ovary; OS, ovary-specific genes within the pistil; PD, genes down-regulated in *dst* compared to HY pistil; PNV, genes expressed in the pistil but not in vegetative tissues; PS, pistil-specific genes in RGAP; PSP, plumose stigma-preferential genes; SP, stigma-preferential genes within the pistil; SS, stigma-specific genes within the pistil

It was reported that 665 probe sets were preferentially expressed in the plumose stigma throughout the rice [49]. When these probe sets were sent to the RGAP (http://rice.plantbiology.msu.edu/), we received 537 plumose stigma-preferential genes (PSP). Among the genes, 468 (87.15%) were preferentially expressed in the stigma, and 47 were specifically expressed in the stigma (Fig. 5a, Additional file 13: Table S10).

Because there is no stigma in the pistil of the *dst* mutant (also called the *dst* ovary), the stigma-specific genes may be theoretically down-regulated in the *dst* pistil (ovary) compared to the HY pistil. To verify the stigma-specific genes, we compared the HY pistil and the *dst* pistil. The results showed that 393 genes were down-regulated in the *dst* ovary compared with the HY pistil (Additional file 4: Figure S2B). Among these candidate genes, we determined only 37 stigma-specific genes within the pistil (Fig. 5b, Additional file 10: Table S8).

### Identification of ovary-/stigma-specific genes during the rice life cycle

During the sexual reproduction process, many genes are expressed in a certain tissue or in a certain type of cells at a certain time. If the temporal and spatial expression pattern is changed, then an abortion or an abnormal seed appears. Many research studies, on genes such as ZmEA1, have provided validation for these findings [48].

Thus, to identify ovary/stigma-specific genes during the life cycle of rice, we downloaded the transcriptome expression data for rice genes from the RGAP (http://rice.plantbiology.msu.edu/expression.shtml). We chose expression data from tissues including a 20-day-old leaf, a 14-day-old shoot, a seedling at the four-leaf stage, an anther, a pistil before pollination, a seed at 5 days after pollination, a seed at 10 days after pollination, an embryo at 25 days after pollination and endosperm at 25 days after pollination. The threshold at which genes were considered to be “expressed” in a tissue was when their FPKM values were not zero in that tissue. We found 159 pistil-specific genes (PS) and 702 pistil-expressed genes that were not expressed in vegetative tissues (PNV) (Fig. 5c). Among the pistil-specific genes, 7 were specifically expressed in the stigma, whereas 45 genes were specifically expressed in the ovary. Additionally, we found 41 stigma-specific genes and 75 ovary-specific genes that were only expressed in reproductive tissues (Fig. 5c, Additional file 14: Table S11).

For the 655 stigma-specific genes that were not included in the PNV, we investigated all their expression in all the tissues including the leaf, shoot, seedling, panicle, anther, pistil and seed (Fig. 5c). The heat map showed that most of the genes were preferentially expressed in the anthers and panicles after they emerged from the leaf sheath (Additional file 15: Figure S4).

### Identification of embryo sac-preferential/specific genes within the pistil

The embryo sac consists of four types of cells that are embedded in the ovary, and it is difficult to investigate. To obtain the candidate genes that are specifically expressed in the embryo sac, we compared the *dst* ovary without the embryo sac to the HY ovary, and we obtained 385 down-regulated genes in the *dst* ovary (Additional file 4: Figure S2C, Additional file 16: Table S12). In 2013, Anderson et al. isolated egg cells, sperm cells and vegetative cells from rice and detected their gene expression by using deep-sequencing technology. They found that 27417 genes were expressed in the egg cells (the threshold, or the point at which the gene that was believed to be expressed in the egg cell, is that when the TPM values were not zero in any biological replicate of the egg cell) [51]. In our study, among these down-regulated genes in the *dst* ovary compared with the HY ovary, 371 (96.4%) genes were expressed in egg cells (Fig. 5d, Additional file 12: Table S12), and the remaining genes may be expressed in other cells of the embryo sac.

As mentioned above, the genes for the embryo sac (female gametophyte)-specific expression belonged to two types of gene sets, that is, the ovary-specific genes and down-regulated genes in the *dst* ovary compared with the HY ovary. Therefore, using Venny analysis, we obtained 122 ovary-specific genes that were down-regulated in the *dst* ovary, suggesting that they may be embryo sac-specific expression genes in the pistil (Fig. 5e, Additional file 17: Table S13). However, we did not know the exact and detailed expression pattern of the genes in the pistil. Thus, we called them genes “embryo sac-preferential/specific genes”. Based on the biological process, these genes were overrepresented in 11 terms, and the top three included the “cell cycle”, “cellular component organization”, and the “DNA metabolic process” (Additional file 12: Figure S3C).

### The expression pattern of embryo sac-preferential/specific genes

**Fig. 6:**
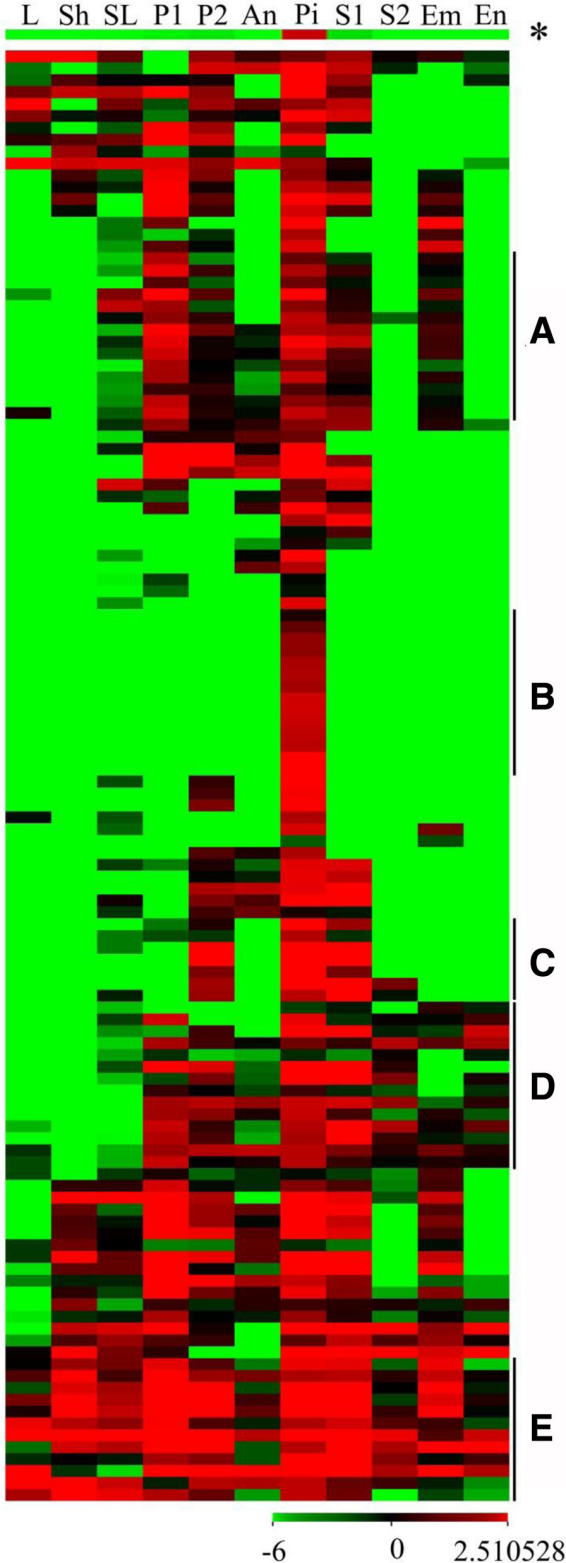
The expression pattern of embryo sac-preferential/specific genes. Next generation sequencing transcriptome data downloaded from the Rice Genome Annotation Project was used for cluster display. Every row represented a gene and every column represented a tissue (indicated at the top of each column). The genes with similar expression patterns are divided into five clusters: **a** (preferentially expressed in P1, P2, Pi and S1), **b** (only in Pi), **c** (preferentially expressed in P2, Pi and S1), **d** (expressed in reproductive tissues but not or weak in vegetative tissues), E (expressed in all tissues). The star represented the general expression levels of all genes. The color represented average log (FPKM values + 0.000001) and the color scale was shown at the bottom. L, 20-day-old leaf; Sh, 14 day-old shoot; SL, seedling at four-leaf stage; P1, panicle before emerging from the sheath of the flag leaf; P2, panicle after emerging from the sheath of the flag leaf; An, anther; Pi, pistil before pollination; S1, seed at 5 days after pollination; S2, seed at 10 days after pollination; Em, embryo at 25 days after pollination; En, endosperm at 25 days after pollination

To determine whether embryo sac-preferential/specific genes within the pistils were only expressed in the pistil, a heat map was drawn. The results showed that all the genes were expressed in the pistil, and most were preferentially expressed (Fig. 6). The general expression pattern showed that the embryo sac-preferential/specific genes were dominantly expressed in the pistils with weak expression in the seeds at the early stage and in the panicles. This finding implied that the embryo sac-preferential/specific genes may play an important role during the development of pistils and seeds. A total of 14 genes were abundantly expressed in the seedlings, panicles, pistils, and seeds (Fig. 6a, Additional file 18: Table S14). Interestingly, 14 genes were found to be present only in the pistils, although some exhibited relatively weak expression (Fig. 6b, Additional file 18: Table S14), suggesting that these genes may play an essential role in the pistil. Similarly, 7 genes were preferentially expressed in the panicles after their emergence from the sheath of the flag leaf, pistils, and seeds during the early stage (Fig. 6c, Additional file 18: Table S14). Additionally, 14 genes were abundantly expressed in reproductive tissues but were not expressed or were weakly expressed in the vegetative tissues (Fig. 6d, Additional file 18: Table S14). Moreover, we found that 12 genes were widely expressed in most organs and tissues (Fig. 6e, Additional file 18: Table S14).

### Homologs of embryo sac-specific genes that function in the pistil

The female gametophyte is indispensable for sexual reproduction in plants. Many embryo sac-specific genes have been isolated from *Arabidopsis*, and some play essential roles [61]. Theoretically, the rice homologs of these genes may be expressed to a high degree in the embryo sac and down-regulated in the ovary of the mutant *dst* compared with the HY ovary if they have similar functions. Because many genes are conserved in different types of plants, a number of known homologous genes have been identified, and they exhibit a similar expression pattern and biological function. Therefore, we identified the rice homologs of *Arabidopsis* functional genes and found that some were down-regulated in *dst*.

**Fig. 7:**
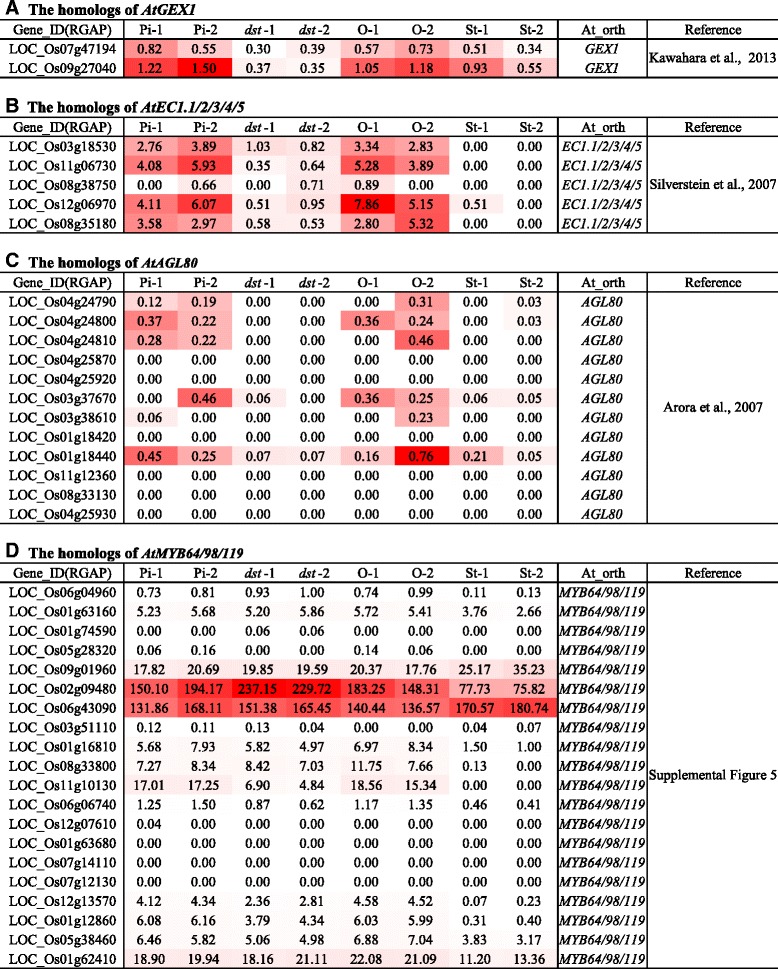
Heat map of rice candidate genes homologous to *Arabidopsis* embryo sac-specific functional genes. Numbers in cells represented FPKM values derived from RNA-seq of HY pistils (Pi), *dst* ovaries (*dst*), HY ovaries (O) and HY stigmas (St) in duplicate. At_orth, Orthology in *Arabidopsis*

AtGEX1 (Gamete-expressed 1) is a plasma membrane protein that plays versatile roles in the development of male and female *Arabidopsis* gametophytes [62]. Using BLASTP searches, we identified two genes (LOC_Os07g47194 and LOC_Os09g27040), and we determined that they were down-regulated in the *dst* mutant compared with the HY ovary (Fig. 7a). Recently, the cysteine-rich proteins (EC1s) were found to be expressed specifically in the egg cells, and they are redundant in male–female gametophyte interactions [63]. There are five members of the ECA1 family in rice (Early Culture Abundant 1), and they are called *ECAGL1/2/3/4/5* [64]. Four of these genes were down-regulated in *dst* compared with the HY ovary, and three of the four genes exhibited an ovary-specific expression pattern (Fig. 7b). Thus, we inferred that these ECA-like proteins may be embryo sac-specific genes that perform similar roles in rice, except for LOC_Os08g38750.

Female gametophyte development is regulated by a complicated gene network that primarily includes transcription factors and their targets. Thus, it is important to identify transcription factors and reveal their functions in female gametophyte development. In *Arabidopsis*, six known transcription factors (AtAGL23/61/80 and AtMYB64/98/119) are specifically expressed in the embryo sac, and they are involved in the development of the female gametophyte [23, 46, 47, 65, 66]. Among the twelve homologs of AtAGL80, six genes were preferentially expressed in the HY ovary and down-regulated in *dst*, but the other six were not expressed in all the tissues (Fig. 7c). In addition, in *Arabidopsis*, there were three MYB transcription factors known as AtMYB64/98/119, which were clustered together (Additional file 19: Figure S5). We identified twenty homologous MYB genes in rice, and LOC_Os11g10130 was significantly decreased in *dst* and specifically expressed in the HY pistil (Fig. 7a, d), suggesting that the gene was embryo sac-preferential/specific. Additionally, the expression of the other five homologs (LOC_Os01g16810, LOC_Os06g06740, LOC_Os12g13570, LOC_Os01g12860 and LOC_Os05g38460) was lower in *dst* than in the HY ovary (Fig. 7d). Moreover, the three MYB transcription factors (LOC_Os02g36890, LOC_Os04g38740 and LOC_Os03g38210) were down-regulated in *dst* compared with the HY ovary (Additional file 19: Figure S5, Additional file 17: Table S13).

### Confirmation via qRT-PCR

**Fig. 8:**
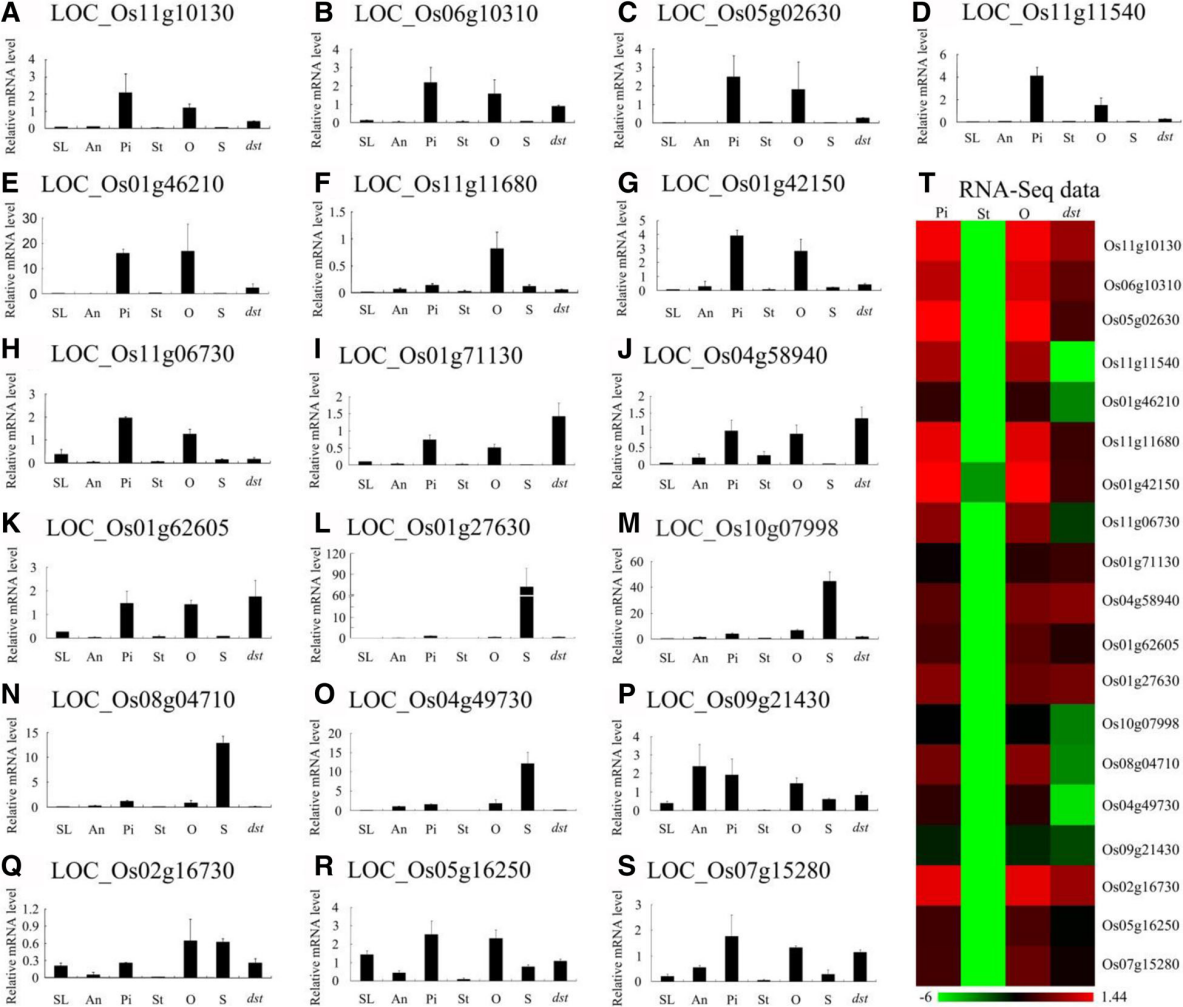
Real-time PCR for confirmation of the 19 representative ovary-specific genes within the pistil. **a**-**s**, the expression pattern of 19 representative ovary-specific genes by qRT-PCR; (**t**), the heat map of the RNA-seq expression levels. SL, 2-weeks-old seedling; An, Anther; Pi, Mature pistil; St, Mature stigma; O, Mature ovary; S, 5 DAP seed; *dst*, *dst* ovary

To verify the expression pattern of these genes in rice and the results of the RNA-Seq, we performed qRT-PCR with RNA from the seedlings (SL), anthers (An), pistils (Pi), stigmas (St), ovaries (O), and seeds (S) from “Hwayoung”, and the ovaries from *dst* (*dst*). Of the 1257 ovary-specific genes, 19 genes were chosen for confirmation, and the results were similar to the results of the RNA-Seq data (Fig. 8a-t). Interestingly, five of the 19 genes (26.3%) exhibited a pistil and ovary-specific expression pattern throughout the plant and were down-regulated in *dst* (Fig. 8a-e). Thus, we predicted that these genes are probably expressed in the embryo sac and play an essential role in the reproductive process, especially LOC_Os11g10130, which may be the homolog of AtMYB64/98/119 (the purple region in Additional file 19: Figure S5). The other six genes were preferentially expressed in the pistils and ovaries, but they showed low expression in the other tissues (Fig. 8f-k). Moreover, four genes were preferentially expressed in the seeds, which implied that the genes may play a more important role in seed development (Fig. 8l-o). The remaining four genes were expressed in all the tissues except in the stigma (Fig. 8p-s). In addition, nine of the 19 genes belonged to the embryo sac-preferential/specific genes and were down-regulated in *dst*, and they were specifically or preferentially expressed in the ovaries and pistils, except LOC_Os08g04710 (Fig. 8a-d, f-h, n, q).

**Fig. 9:**
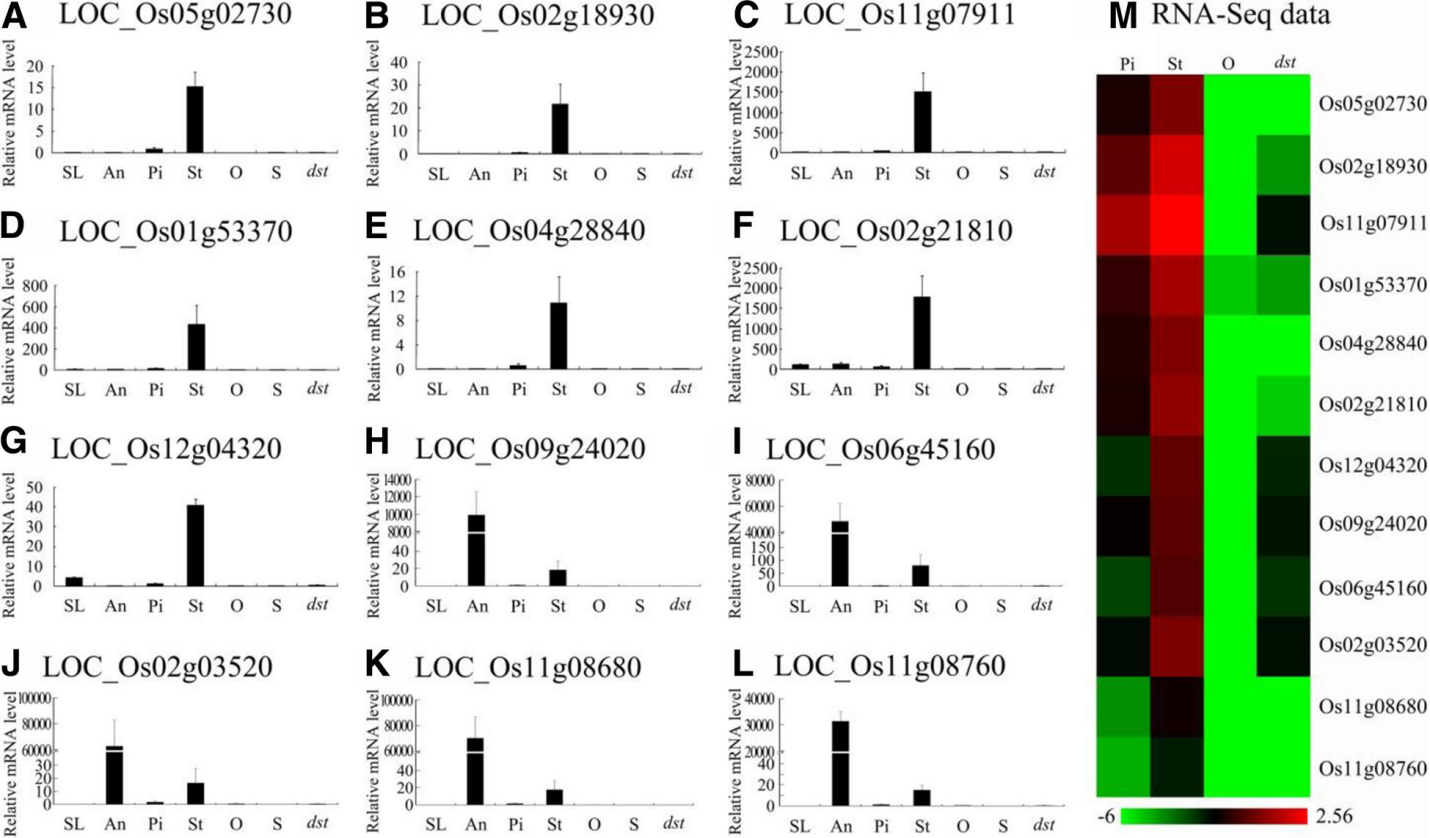
Real-time PCR for confirmation of the 12 representative stigma-specific genes within the pistil. **a**-**l**, the expression pattern of 12 representative stigma-specific genes by qRT-PCR; (**m**), the heat map of the RNA-seq expression levels. SL, 2-weeks-old seedling; An, Anther; Pi, Mature pistil; St, Mature stigma; O, Mature ovary; S, 5 DAP seed; *dst*, *dst* ovary

As Fig. 5b shows, there were 703 stigma-specific genes, and 37 of them were down-regulated in *dst* compared with the HY pistil. To confirm these results, we detected the expression of 12 genes, and we found that their expression was similar to the results from the RNA-Seq data (Fig. 9a-m). The result showed that five genes were expressed only in stigmas and pistils (Fig. 9a-e). Two genes were preferentially expressed in the stigmas (Fig. 9f, g), whereas five genes were predominantly expressed in anthers compared with stigmas, which implied that these genes may play vital roles in the stigma or anther development or in the interaction between pollen grains and stigmas (Fig. 9h-l).

### Confirmation using mRNA *in situ* hybridization

**Fig. 10:**
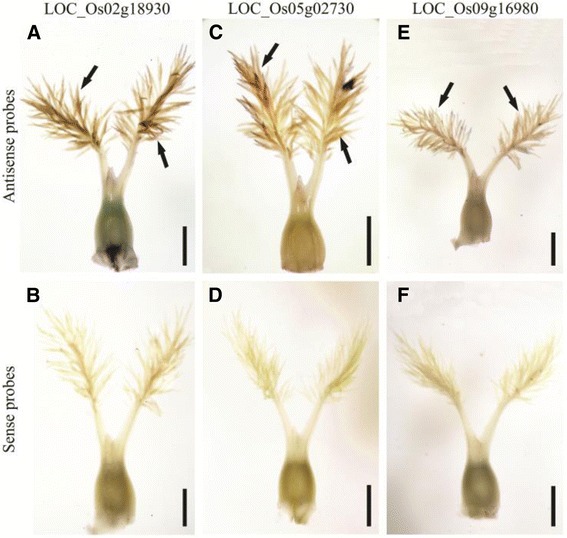
The confirmation of stigma-specific genes by whole mount *in situ* hybridization. We verified three genes, including LOC_Os02g18930 coding a putative calcineurin B (**a**-**b**), LOC_Os05g02730 coding a homeobox domain containing protein (**c**-**d**), and LOC_Os09g16980 coding a wall-associated protein kinase OsWAK86 (**e**-**f**). Bar was 500 μm. The hybridization signals (pointed with black arrows) are shown in dark brown and the signals of all genes were only present in stigma papilla cells

To further verify the stigma-specific genes, three genes, including a putative calcineurin B (LOC_Os02g18930), a homeobox domain-containing protein (LOC_Os05g02730), and a wall-associated protein kinase, OsWAK86 (LOC_Os09g16980), were selected for RNA whole-mount *in situ* hybridization. Mature pistils that were collected at the time just before pollination were used for the hybridizations. The results showed that all three genes exhibited a signal in the stigma papilla cells but not in the ovary (Fig. 10). Interestingly, the former two genes were found to only be expressed in the stigma throughout the rice life cycle (Fig. 8), implying that they may be associated with pollination success. Besides, we observed the expression of embryo sac-preferential/specific genes LOC_11g10130 (homologous gene of AtMYB64/98/119) through *in situ* hybridization and found that it was present in antipodal cell but not in the other part of the ovary (Additional file 20: Figure S6A-B).

### Novel protein-coding genes

When the RNA-Seq reads were mapped onto the rice genome and transcriptome, we found that many reads were enriched in some regions, but far away from the known genes. To investigate these previously unannotated regions, all of the clean reads were mapped onto the rice genome and compared to the transcriptome from the RAP-DB (Rice Annotation Project Database; http://rapdb.dna.affrc.go.jp/download/irgsp1.html). In total, 1537 transcripts of 797 genes were found more than 200 bp away from any annotated gene. After the transcripts with CPC ≤ 0 were removed, 1097 transcripts from 571 genes were obtained (data not shown). Using a comparison with the transcriptome from the RGAP 7.0 version, the transcripts that were found to overlap with any known gene were removed again. Finally, we identified 201 transcripts of 102 novel genes. In these genes, there were 33 genes with 1000 or more supported reads and 16 genes with 2000 or more supported reads (Table 2, Additional file 21: Table S15).

**Table 2:** The novel protein-coding genes in rice

	Number of transcripts	Number of genes
Total	201	102
100 ≥ the supported reads >0	18	17
1000 ≥ the supported reads >100	98	52
2000 ≥ the supported reads >1000	42	17
the supported reads >2000	43	16

**Fig. 11:**
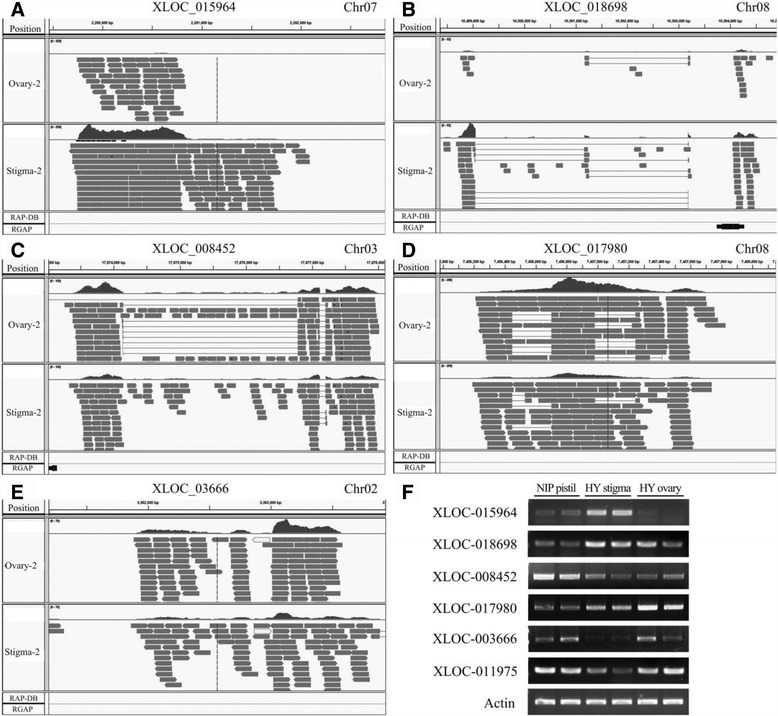
The novel protein-coding genes. **a**-**e**, Screen captures from IGV software showed regions of the putative novel genes outside the previously annotated genes. Every screen capture was divided into 5 tracks. From top to bottom, these tracks were as follows: positions at the chromosome, read alignments in the second sample of HY ovary, read alignments in the second sample of HY stigma, the gene model annotations from the Rice Annotation Project Database (RAP-DB), the gene model annotations from the Rice Genome Annotation Project. The positions of the five regions were as follows: **a**, chr07: 2279460–2282852; **b**, chr08: 10498364–10504912; **c**, chr03: 17873014–17878106; **d**, chr08: 7455980–7458153; **e**, chr02: 3561191–3563938. In the second and third track, each track was divided into two smaller tracks: the gray graphs of the upper track indicated the number of reads that map at the positions indicated in the coordinates track; the gray boxes were the reads, and thin lines indicated reads mapping across introns. In the fourth and fifth tracks, the black boxes were the previously annotated genes. **f**, Confirmation of the putative novel genes using semi-quantitative RT-PCR. NIP, Nipponbare; HY, Hwayoung

To view the read alignments and splicing patterns, the Integrative Genomics Viewer (IGV) was downloaded from its website (http://software.broadinstitute.org/software/igv/download) and applied here [67]. The read alignments showed that some novel genes were preferentially expressed in the stigma or ovary (Fig. 11a-e).

Furthermore, a *t*-test was performed between the HY stigma and HY ovary. The results showed that 2 novel genes were preferentially expressed in the stigma, whereas there were 3 novel genes in the ovary of those genes with 1000 or more supported reads (P < 0.05; Additional file 21: Table S15), except XLOC_015964. Moreover, to determine whether there was a difference in the expression of some genes between varieties, we tested the RNA levels from the Nipponbare pistil, HY ovaries, and HY stigmas using semi-quantitative RT-PCR (Fig. 11f). All six novel genes were present in both Nipponbare and Hwayoung, suggesting that they are widely present in different varieties of *japonica*. XLOC-015964 was preferentially expressed in the stigma, whereas the other three genes (XLOC-017980, XLOC-003666 and XLOC-011975) were expressed in the ovaries (Fig. 11f). The expression of the other two genes (XLOC-018698 and XLOC-008452) exhibited no significant differences between the rice ovary and stigma (Fig. 11f). These novel genes could be valuable for the supplementation and perfection of the existing rice transcriptome.

## Discussion

### Identification of stigma-preferential/specific genes within the pistils

Despite the existence of many research reports about rice reproduction, little is known about the molecular mechanisms of the stigma-development and pollination. Thus, to dissect the molecular basis of these mechanisms in rice, we performed RNA-Seq, and we identified 3531 stigma-preferential genes. Compared with the ovary, the stigma exhibited a distinct genome-wide expression pattern (Fig. 4). The transport-, localization-, membrane-, communication-, secretion- and pollination-related genes were significantly enriched in the stigma, while genes related to the extracellular region were not (Fig. 4a, Additional file **7**: Table S5). The results showed that the rice stigma, whose transmitting tracts were made up of several compactly arranged cell layers around the vascular bundle, might perform its function primarily through the membrane, and it was different from the wet stigma in tobacco, which is composed of transmitting tissue and papilla cells with vigorous secretion activity [28, 68]. Thus, the wet stigma primarily performed its role through the extracellular matrix. In tobacco, floral-transmitting tissue-specific arabinogalactan proteins [transmitting tissue specific proteins (TTS)] were located in the extracellular matrix of the transmitting tissue. TTS attracted the pollen tubes, stimulated their growth, and were then deglycosylated to form a glycosylation gradient in the stigma and style [28, 69]. Conversely, dry stigmas disturbed the adhesion of incompatible pollen grains primarily via membrane-related receptors in the stigma. For example, *Brassica* recognized the incompatible pollen grains primarily through SRK and its downstream pathways [6]. Based on the GO analysis, we also found that the genes for secondary metabolism were overrepresented in the stigma. This GO term was also enriched in the stigma-specific genes within the pistil, and many pathways related to the secondary metabolism were enriched in the KEGG analysis of stigma-preferential genes within the pistil, although few have been studied in rice (Fig. 4a, Additional file 12: Figure S3A, Additional file 8: Table S6). In *Arabidopsis*, a type of pistil extract called azadecalin was isolated, and it could stimulate pollen germination. Azadecalin-like molecules also stimulate pollen germination [18]. Thus, there may be some secondary metabolites, such as azadecalin-like molecules, that play essential roles during fertilization. To date, many stigma-specific genes have been found that are involved in the fertilization process of many species. For example, a cysteine-rich protein called *STIG1* (stigma-specific protein 1) was found to be specifically expressed in the stigma, and it controls exudate secretion in tobacco and petunias. *PrsS* (*Papaver rhoeas* stigma S determinant), a stigma-specific gene, is involved in the S-specific inhibition of incompatible pollen grains, which is similar to SRK in *Brassica* [6, 17, 70]. However, little is known about stigma development and pollination in rice. Thus, in our study, 703 stigma-specific genes were identified within the pistil, and 47 of them were preferentially expressed in the plumose stigma, whereas 7 genes were only expressed in the stigma (Fig. 5). Interestingly, among these genes, we found two cysteine-rich peptides (LOC_Os02g55810 and LOC_Os02g55510) and two S-locus-related genes (LOC_Os01g48000 and LOC_Os01g48040) (Additional file 8: Table S8). These genes may participate in the stigma development or inhibition of incompatible pollen grains. Our study sets the foundation for gene function research in relation to the stigma and pollination.

### Gene conservation and divergence between rice and *Arabidopsis*

Thus far, many embryo sac-specific genes have been shown to be involved in the development of normal embryo sacs and the success of double-fertilization in *Arabidopsis* [59]. In rice, only two embryo sac-specific genes have been identified, but their functions are still unknown [50]. As described above, there was no embryo sac in most of the *dst* ovaries (Fig. 1), and the two known rice embryo sac-specific genes were down-regulated in the rice *dst* ovary compared with the HY ovary (Additional file 1: Table S1). Thus, the genes that were down-regulated in the rice *dst* ovary compared with the HY ovary may be present in the embryo sacs. Additionally, many homologous genes were down-regulated in the rice *dst* ovary, implying that they have a similar expression pattern in rice (Fig. 7). Among the different species, many genes with a similar expression pattern and high homology, such as *OsFZP* and *ZmBD1*, play similar roles during the transition of the spikelet meristem to a floret meristem, and *OsDMC1* and *AtDMC1* also perform similar functions during homologous pairing [71–75]. Hence, we concluded that some of these homologous rice genes may be indispensable for the development of the embryo sac and fertilization, and there may be similar molecular mechanisms between rice and *Arabidopsis* during the reproductive process.

There were also partially or highly different mechanisms between rice and *Arabidopsis* with the evolution of the species. For example, the knockout of AtWUSCHEL resulted in defective shoot, flower, and fruit development, while the putative rice ortholog of AtWUSCHEL, which is called OsTAB1 (TILLERS ABSENT1), only regulated the initiation of axillary meristems with no effect on the shoot apical meristems [76]. In *Arabidopsis*, mutations in the FIS complex genes (MEA, FIS2, FIE and MSI1) resulted in autonomous endosperm development without fertilization, and their seed developments were arrested at the heart embryo stage [77, 78]. In rice, there were no MEA and FIS2 genes, but two FIE orthologs, FIE1 and FIE2 are present. When FIE1 and FIE2 were knockout or knockdown, plants in rice failed to show autonomous central cell proliferation without fertilization [78–80]. In correspondence with function divergences, their expression patterns were also different. In our study, both OsFIE1 (LOC_Os08g04290) and OsFIE2 (LOC_Os08g04270) were not down-regulated in the rice *dst* ovary compared with the HY ovary, and many other homologous genes were also not down-regulated in the rice *dst* ovary (Additional file 22: Figure S7A). There are five lysophosphatidyl acyltransferases (LPATs) in *Arabidopsis* and three in rice. Among them, AtLPAT2 is specifically expressed in the embryo sac, and it is essential for the development of the embryo sac [81]. However, none of the three rice genes were down-regulated in *dst*, suggesting that they may play different roles (Additional file 22: Figure S7B). Similarly, an *Arabidopsis* central cell-specific gene, AtCCG (central cell guidance), acts as an essential transcription regulator for fertilization [24], whereas its homolog in rice is abundantly expressed in the *dst* ovary and wild type HY ovary with no significant difference (Additional file 22: Figure S7C). Moreover, a synergid cell-specific putative glucosyl phosphatidylinositol-anchored protein called AtLORELEI controls the release of sperm cells in *Arabidopsis* [82]. Using a BLASTP search, we found six homolog genes of AtLORELEI in rice. None of them were down-regulated in the *dst* mutant compared with the HY ovary, implying that the genes may not have functions similar to those in *Arabidopsis*. Interestingly, five of the six were preferentially expressed in the rice stigma, suggesting that they may play an essential role in stigma development or during pollination (Additional file 22: Figure S7D). To confirm our results, we use *in situ* hybridization to detect the three gene expression of LOC_11g41900 (homologous gene of AtLPAT2), LOC02g48980 and LOC_06g19990 (homologous gene of AtLORELEI). All the three genes were present in the entire ovary, not restricted in the embryo sac (Additional file 20: Figure S6C-H). All of the above rice genes from *Arabidopsis* homologs may perform different functions, implying that there is a divergent mechanism of individual development between rice and *Arabidopsis*.

### Stigma-specific genes may show weak levels of expression

In comparing the stigma and ovary in Hwayoung rice, we identified 703 genes that were specifically expressed in the stigma. Theoretically, the stigma-specific genes would be down-regulated in the mutant *dst* ovary (no sigma) compared with the HY pistil. Unexpectedly, there were only 37 (5.3%) stigma-specific genes that were down-regulated in the mutant *dst* ovary (Fig. 5b). The analysis of the expression data showed that the FPKM values of most of the stigma-specific genes were very low in the stigma, and they were not greater than 10 (Additional file 10: Table S8). Among the stigma-specific genes, with the exception of the genes that were down-regulated in the mutant *dst*, they exhibited weaker expression (Additional file 23: Figure S8). In *Arabidopsis*, the 71 genes that were down-regulated in the mutant *dif* were screened using microarrays [45]. However, *AtMYB64/119* and *AtAGL61*, which were confirmed to be female gametophyte-specific genes in the following research, were not included because of their weak expression [46, 47, 83]. This result suggested that many cell-type-/stigma-specific genes were expressed weakly and could not be screened out by comparing the wild-type with the mutant gene using high-throughput analysis. Therefore, multiple techniques are required.

### Analysis of the embryo sac-preferential/specific genes was conducive to investigating the gene function and regulation network in the female rice gametophyte

In our results, the expression of 122 genes was dependent on the existence of an embryo sac and may be specifically expressed in the ovary compared with the stigma in rice (Fig. 5e). Using reverse-genetic approaches, including T-DNA insertion and small RNA silencing, the functions of the genes could be investigated further, and some had to be knocked out/down together because they may play a redundant role. In *Arabidopsis*, both *MYB64* and *MYB119*, which exhibit a female gametophyte-specific expression pattern, redundantly regulated the FG5 (Female Gametophyte 5) transition. The single mutants *MYB64* or *MYB119* showed no apparent defect, but the double mutant, *myb64myb119*, failed to initiate the FG5 transition [47]. Similarly, the five egg cell-specific genes known as the *EC1s* [63] and the five synergid cell-specific genes known as *LUREs* [30] also performed redundant functions in *Arabidopsis*. In our study, we found that many embryo sac-preferential/specific genes may also have redundant functions, such as the *Arabidopsis EC1s’* rice homologs, the *ECA1*-like genes.

Furthermore, we could construct promoter::GUS/GFP vectors, transform them into rice plants, and obtain lines containing the female gametophyte-specific marker for future research on female gametophyte development. For example, the four types of cells in the female gametophyte could be isolated and collected separately using a cell-sorting approach, and more cell type-specific genes could be identified. Moreover, with the help of the promoter:: GFP lines, we could identify rice mutants that have abnormal cell fates. Additionally, the promoters of these cell-specific genes could be used to identify the *cis*-regulatory elements, which would help us to investigate the gene regulatory function and network involved in female gametophyte development. In conclusion, these embryo sac-preferential/specific genes could be used as starting points for research on female gametophytes in rice.

### More accurate method for gene identification and validation

With technological progress, the rice genome sequence and transcriptome became more accurate and more detailed. In 2002, the draft sequences of the rice genome, including *japonica* and *indica*, were completed using whole-genome shotgun sequencing. More than 32,000 genes have been predicted via assembled sequences, covering 93% of *japonica* and 92% of *indica* rice [84, 85]. Recently, high-throughput sequencing technology has attracted the attention of scientists because it has the advantages of having a low cost, good repeatability, and high-throughput capacity. By using the high-throughput sequencing technology, the Nipponbare genome assembly was updated, and the error rate was only approximately 0.15 per 10,000 nucleotides in the original IRGSP assembly [52]. With the help of a new reference genome, 1240 loci from the RGAP 6.1 annotation set were thrown out from the RGAP 7.0 annotation set and 4993 loci from the RAP annotation on the IRGSP build 5 genomes were excluded in the new release.

Moreover, in animals and *Arabidopsis*, high-throughput transcriptome sequencing has been used to explore alternative splicing and novel genes to simultaneously discover transcripts and estimate their abundance [86, 87]. In rice, high-throughput transcriptome sequencing has primarily been used to detect gene expression, but almost none has been used for detecting novel genes. In our study, 102 novel protein-coding genes were identified, and 6 of them were verified in two Hwayoung and Nipponbare cultivars (Table 2, Fig. 11). Thus, we suggest that there are more novel genes in other rice tissues besides the pistil. Thus, using high-throughput transcriptome sequencing data in different rice tissues, the novel genes were revealed and verified more accurately and effectively.

## Conclusions

In this study, we explored numerous stigma-preferential genes within the pistil through a comparison between the ovary and stigma by using RNA-Seq, and we found that the transport-, localization-, membrane-, communication-, secretion- and pollination-related genes were significantly enriched in the stigma through GO analysis, indicating that rice stigma may perform its function primarily through the membrane and that it was different from the wet stigma. Among these genes, many were specifically expressed in the stigma and verified by qRT-PCR and *in situ* hybridization, implying that they may play important roles in stigma development and pollination. In addition, many embryo sac-preferential/specific genes were found through the comparison between the wild ovary and *dst* ovary (without embryo sac). Most of them were preferentially expressed in pistils and may only be expressed in female gametophytes, suggesting that they may have essential roles in female gametophyte development and fertilization and could be used for investigating gene functions and molecular mechanisms. Moreover, we found that many “reads” were clustered far away from the annotated genes; we identified some novel protein-coding genes in the rice pistils, suggesting that the transcriptome data would serve as a valuable resource for identifying rice genes to supplement and perfect the existing transcriptome in rice. Therefore, these studies provided an important foundation for investigating the pollination and fertilization mechanisms in rice stigmas and female gametophytes.

## Methods

### Plant materials

The Rice plants (*Oryza sativa* L. ssp. japonica, cultivar ‘Hwayoung’, HY; cultivar ‘Nipponbare’, NIP), for RNA-sequencing and verification by semi-quatative RT-PCR, qRT-PCR and RNA *in situ* hybridization, were grown in green house at Wuhan University. The HY seedlings (SL) were collected after seeds were germinated and grown for 2 weeks. After the panicles emerged from the sheath of flag leaf, the samples of un-pollnated pistils (Pi), ovaries (O), stigmas (St), and anthers (An) were dissected from Hwayoung, meanwhile, the ovaries of *dst* (*defective stigma*) mutant, obtained from RISD DB (Rice T-DNA Insertion Sequence Database), were dissected before the florets flowering. And pistils from Nipponbare (NIP pistil) were collected after the panicles emerged from the sheath of flag leaf. At last, 5-d seed of HY (S) after the florets flowering were collected.

### Ovary transparency and microscopy observation

The mature florets before flowering were collected, then stained using a modified protocol of the whole-mount eosin B colorant, and observed under confocal laser scanning microscopy according to Zeng’s method [88]. The cleared ovaries were scanned and observed under an Olympus Fluoview 1000 laser scanning confocal microscope with a 535 nm argon laser.

### RNA extraction and sequencing analysis

Total RNAs for RNA-sequencing from the pistils, ovaries, stigmas of wild type plants as well as ovaries of *dst* were extracted using Trizol reagent (Invitrogen, USA) following the manufacturer’s protocol and digested with DNaseI, and then quantified under Nanodrop Spectrophotometer (Nanodrop Technologies, Wilmington, USA) and Agilent 2100 Bioanalyzer (Agilent Technologies, Böbelingen, Germany). After that, the samples were prepared using Illumina’s kit according manufacturer’s recommendation, and sequenced on the Illumina sequencing platform (HiSeqTM 2000) by the Shanghai Oebiotech Corporation. And 125 bp paired-end reads were generated. Our files containing sequence reads and quality scores were deposited in the Short Read Archive of the National Center for Biotechnology Information (NCBI) [Accession number SRP082242].

### RNA-seq data preprocessing

Raw data (raw reads) of fastq format were firstly processed using the NGS QC Toolkit [89], and reads containing adapter or ploy-N and low quality were removed. All the downstream analyses were based on clean data with high quality (Q30). Then the sequencing reads were mapped to the rice genome (http://rice.plantbiology.msu.edu/pub/data/Eukaryotic_Projects/o_sativa/annotation_dbs/pseudomolecules/version_7.0/all.dir/) using Tophat (http://ccb.jhu.edu/software/tophat/index.shtml), and the mapped reads were assigned to the RGAP 7.0 gene modes using Bowtie2 [52, 90, 91] with default parameters by slightly modified. The FPKM (fragments per kilobase of gene per million) and count value were calculated using eXpress [92].

### Differential expression analysis

Differential expression analysis was performed using the DESeq R package [93]. The FDR (False Discovery Rate) ≤0.05 and fold change ≥ 2 was used as the threshold for significantly differential expression. The hierarchical cluster analysis of samples was also performed using the DESeq R package, and PCA (principal component analysis) was performed using the plotPCA function of the DESeq2 package. The first and second principal components explained 46.2% and 15.1% of variance, respectively. Comparison of GO function categories between ovary- and stigma-preferential genes was calculated using the WEGO online webtool (http://wego.genomics.org.cn/cgi-bin/wego/index.pl) based on Pearson Chi-Square test. The GO terms were removed if any of the expected counts were less than 5 or the p-value was more than the significant level of 0.05 [58]. The other GO and KEGG enrichment analyses of the differentially expressed genes (DEGs) were performed based on hypergeometric distribution.

### Digital expression analysis

Next generation sequencing transcriptome data were downloaded from the Rice Genome Annotation Project (http://rice.plantbiology.msu.edu/expression.shtml). The average value of a gene in one tissue plus 0.000001 was used logarithmic operation. Then, the logarithmic values were used for cluster display with the help of Cluster 3.0 and Treeview software.

### Phylogenetic analysis

BLASTP searches were performed to identify homologous genes using the sequences of AtCCG, AtLORELEI, AtMYB64/98/119, AtGEX1 and secretion-related proteins (GO: 0046903) with E-value = 0.00001. The candidate homologous genes of *AtMYB64/98/119* were aligned in Clustal X (version 1.83) and MEGA 6 using neighbor-joining method (the bootstrap value was 500) [94, 95]. Then, an un-rooted phylogenetic tree was drawn.

### Novel protein-coding gene forecast

The clean reads were mapped onto rice genome using Tophat (http://ccb.jhu.edu/software/tophat/index.shtml). Subsequently, transcripts were assembled using Cufflinks [96], and the short transcripts (the length <180 bp) were removed. Then, we identified the genes that were more than 200 bp away from any IRGSP-1.0_representative gene, and calculated the potential ability of coding protein using Coding Potential Calculator (CPC, http://cpc.cbi.pku.edu.cn/) while CPC ≥ 0 was set as the threshold [97]. Besides, the transcriptome from the RGAP 7.0 version was also compared with the coding genes (CPC ≥ 0), and the genes that did not overlapped with any gene were thought as novel genes.

### Quantitative RT-PCR and semi-quantitative RT-PCR analysis

Except the RNA samples for sequencing, the other RNA was extracted with the RNApure Extraction Kit (Bioteke, Beijing, China) according to the manual of the Kit. The methods of reverse transcription and RT-PCR were performed according to Zhao et al. [98]. The threshold cycle (C_T_) value was automatically calculated by the Bio-Rad CFX Manager 3.1 system software, and the delta-delta Ct method was used to calculate the relative expression levels [99, 100]. The rice *eEF-1α* gene was used as an internal control for quantitative RT-PCR, while *Actin* gene for semi-quantitative RT-PCR [54]. The primers were listed in the Additional file 24: Table S16.

### *In situ* hybridization

Using the DIG RNA labeling kit (Roche), the digoxigenin labeled RNA probes were generated according the manual. The protocol of whole mount *in situ* hybridization was similar to the Hu’s method with some modifications and the protocol of the paraffin section was similar to Brewer’s procedure [101, 102]. The primers used were listed in the Additional file 24: Table S16.

## Additional files

Additional file 1: Table S1. The expression of known tissue-specific genes in the pistil. (XLS 27 kb)
Additional file 2: Table S2. The known genes not to be expressed in the pistil. (XLS 25 kb)
Additional file 3: Figure S1. The saturation analysis. (PDF 314 kb)
Additional file 4: Figure S2. The heat maps of the differential expressed genes (DEGs). (PDF 146 kb)
Additional file 5: Table S3. The ovary-preferential genes within the pistil. (XLS 1022 kb)
Additional file 6: Table S4. The stigma-preferential genes within the pistil. (XLS 1340 kb)
Additional file 7: Table S5. The secretion-related genes. (XLS 40 kb)
Additional file 8: Table S6. The KEGG analysis of stigma-preferential genes within pistil. (XLS 40 kb)
Additional file 9: Table S7. The KEGG analysis of ovary-preferential genes within the pistil. (XLS 34 kb)
Additional file 10: Table S8. The stigma-specific genes within the pistil. (XLS 326 kb)
Additional file 11: Table S9. The ovary-specific genes within the pistil. (XLS 355 kb)
Additional file 12: Figure S3. GO analysis based on biological process. (PDF 186 kb)
Additional file 13: Table S10. The results of comparison of stigma-preferential/specific genes in different studies. (XLS 149 kb)
Additional file 14: Table S11. Stigma-/ovary-specific genes throughout the rice. (XLS 85 kb)
Additional file 15: Figure S4. The expression pattern of stigma-specific genes. (PDF 174 kb)
Additional file 16: Table S12. The genes down-regulated in dst ovary compared to HY ovary. (XLS 126 kb)
Additional file 17: Table S13. The embryo sac-preferential/specific genes within the pistil. (XLS 79 kb)
Additional file 18: Table S14. The expression pattern of embryo sac-preferential/specific genes. (XLS 51 kb)
Additional file 19: Figure S5. A phylogenetic tree of rice genes homologous to AtMYB64/98/119 in the purple region. (PDF 355 kb)
Additional file 20: Figure S6.
*In situ* hybridization. (PDF 317 kb)
Additional file 21: Table S15. The novel protein-coding genes in rice. (XLS 97 kb)
Additional file 22: Figure S7. Heat map of rice candidate genes homologous to some Arabidopsis embryo sac-specific functional genes. (PDF 23 kb)
Additional file 23: Figure S8. The respective expression levels of stigma-specific genes in HY stigma. (PDF 184 kb)
Additional file 24: Table S16. The sequences of primers. (XLS 34 kb)

## Abbreviations

An: Anthers
DAP: Days after pollination
*dst*: *defective stigma*
FPKM: Fragments per kilobase of exon per million mapped reads
GO: Gene Ontology
HY: Hwayoung
NIP: Nipponbare
O: Ovaries
Pi: Pistils
qRT-PCR: Quantitative real time polymerase chain reaction
RAP-DB: Rice Annotation Project Database
RGAP: Rice Genome Annotation Project
S: Seeds
SL: Seedlings
St: Stigmas
