# Supplementary material for: Identification and analysis of the stigma and embryo sac-preferential/specific genes in rice pistils
Source: BMC Plant Biol. 2017 Mar 7;17:60. doi: 10.1186/s12870-017-1004-8 (PMC5341191; doi:10.1186/s12870-017-1004-8)
Supplement: Additional file 3: Figure S1. — The saturation analysis. (PDF 314 kb) [file 12870_2017_1004_MOESM3_ESM.pdf]

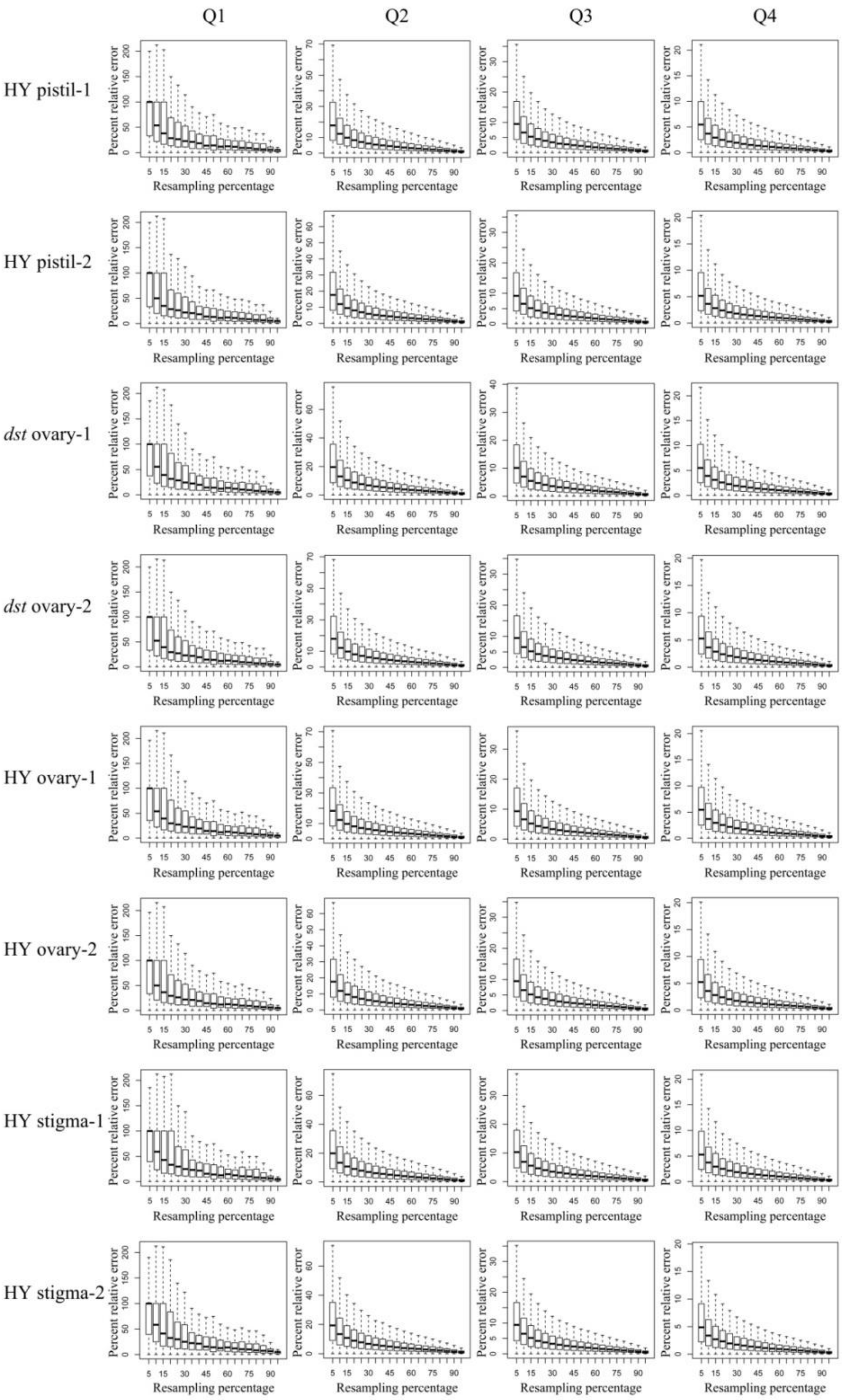

Q1
Q2
Q3
Q4
HY pistil-1
HY pistil-2
dst ovary-1
dst ovary-2
HY ovary-1
HY ovary-2
HY stigma-1
HY stigma-2
Percent relative error
Resampling percentage

Supplemental Figure 1. The saturation analysis. Every row represented a sample and every column represented a cluster of genes with similar expression. The x axis represented the resampling percentage of data and the y axis represented the relative error of the gene expression under the resampling data. Q1, Q2, Q3, Q4 represent different clusters of genes with expression from low to high. All of the genes with different expression showed low relative error when the resampling percentage was more than 90% even if the expression of genes were very low.
