# Supplementary material for: Identification and analysis of the stigma and embryo sac-preferential/specific genes in rice pistils
Source: BMC Plant Biol. 2017 Mar 7;17:60. doi: 10.1186/s12870-017-1004-8 (PMC5341191; doi:10.1186/s12870-017-1004-8)
Supplement: Additional file 4: Figure S2. — The heat maps of the differential expressed genes (DEGs). (PDF 146 kb) [file 12870_2017_1004_MOESM4_ESM.pdf]

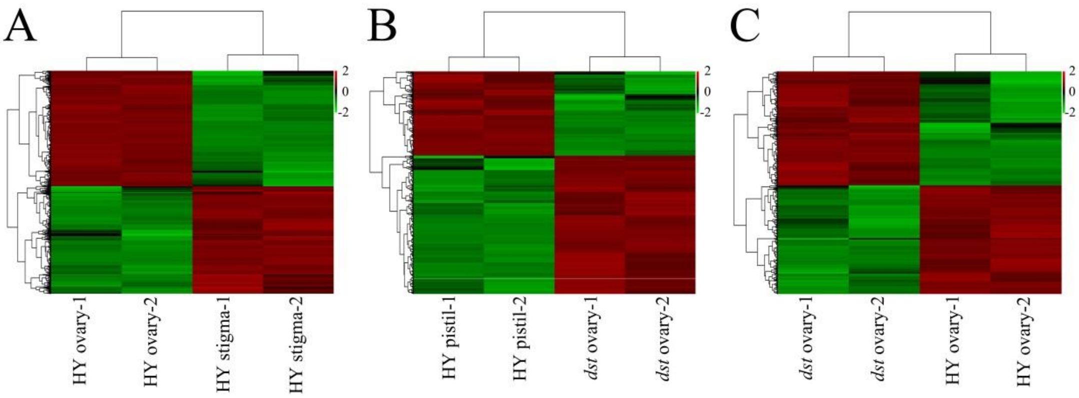

Supplemental Figure 2. The heat map of the differential expressed genes (DEGs). Green and red indicated lower and higher expression, respectively. The color scale was shown in the upper right. A, Clustering and heat map of expression values for DEGs between HY ovary and HY stigma. B, Clustering and heat map of expression values for DEGs between HY pistil and *dst* ovary. C, Clustering and heat map of expression values for DEGs between HY ovary and *dst* ovary.
