# Supplementary material for: Identification and analysis of the stigma and embryo sac-preferential/specific genes in rice pistils
Source: BMC Plant Biol. 2017 Mar 7;17:60. doi: 10.1186/s12870-017-1004-8 (PMC5341191; doi:10.1186/s12870-017-1004-8)
Supplement: Additional file 12: Figure S3. — GO analysis based on biological process. (PDF 186 kb) [file 12870_2017_1004_MOESM12_ESM.pdf]

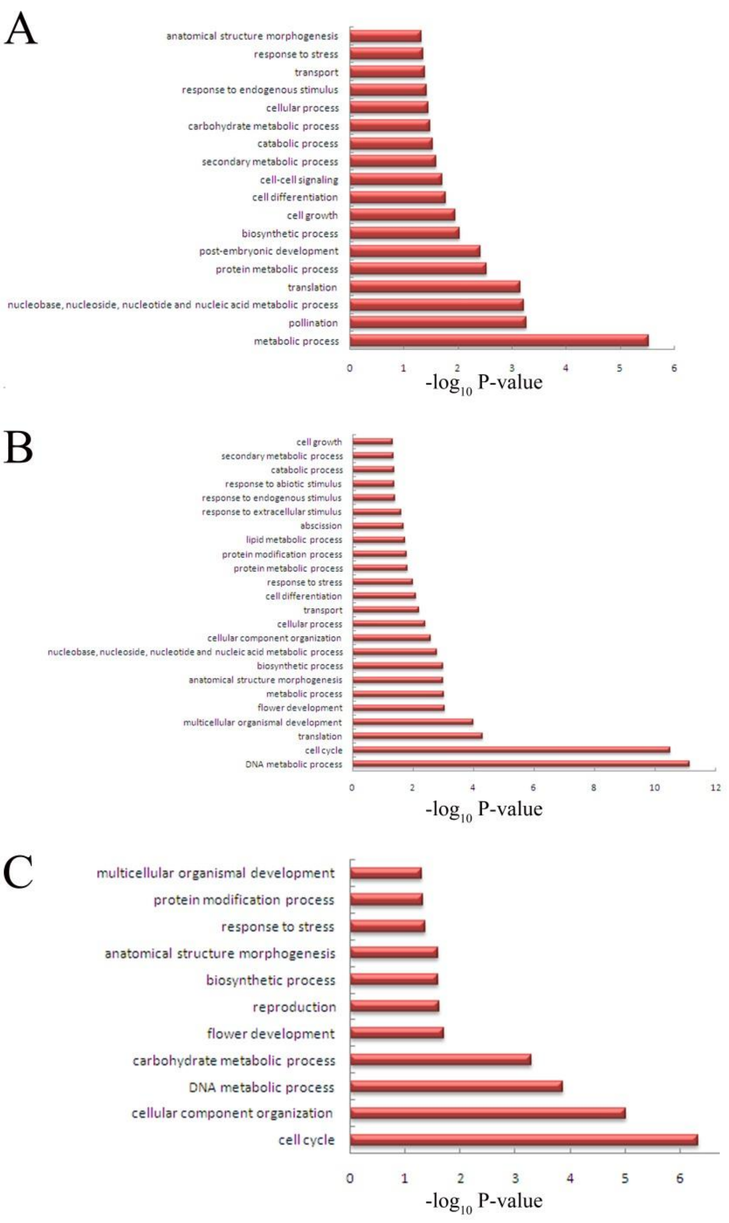

Supplemental Figure 3. GO analysis based on biological process. We identified 703 stigma-specific genes, 1257 ovary-specific genes and 122 embryo sac-dependent genes. Based on biological process, the stigma-specific genes were enriched in 18 GO terms (A), the ovary-specific genes were enriched in 24 terms (B), and the embryo sac-dependent genes were overrepresented in 11 terms (C) ($P < 0.05$).
