# Supplementary material for: Identification and analysis of the stigma and embryo sac-preferential/specific genes in rice pistils
Source: BMC Plant Biol. 2017 Mar 7;17:60. doi: 10.1186/s12870-017-1004-8 (PMC5341191; doi:10.1186/s12870-017-1004-8)
Supplement: Additional file 15: Figure S4. — The expression pattern of stigma-specific genes. (PDF 174 kb) [file 12870_2017_1004_MOESM15_ESM.pdf]

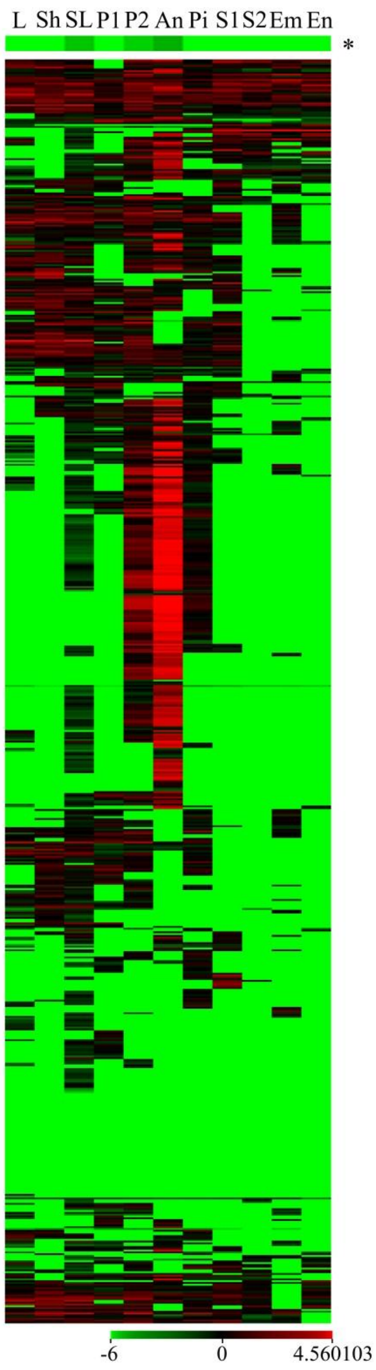

Supplemental Figure 4. The expression pattern of stigma-specific genes. The star represented the general expression of all genes which was preferentially expressed in seedlings, panicles after emerging from the sheath of the flag leaf and anthers. The color represented average log (FPKM values+0.000001) and the color scale was shown at the bottom. L, 20-day-old leaf; Sh, 14 day-old shoot; SL, seedling at four-leaf stage; P1, panicle before emerging from the sheath of the flag leaf; P2, panicle after emerging from the sheath of the flag leaf; An, anther; Pi, pistil before pollination; S1, seed at 5 days after pollination; S2, seed at 10 days after pollination; Em, embryo at 25 days after pollination; En, endosperm at 25 days after pollination.
