# Supplementary figures and images for: Identification and analysis of the stigma and embryo sac-preferential/specific genes in rice pistils
Source: BMC Plant Biol. 2017 Mar 7;17:60. doi: 10.1186/s12870-017-1004-8 (PMC5341191; doi:10.1186/s12870-017-1004-8)

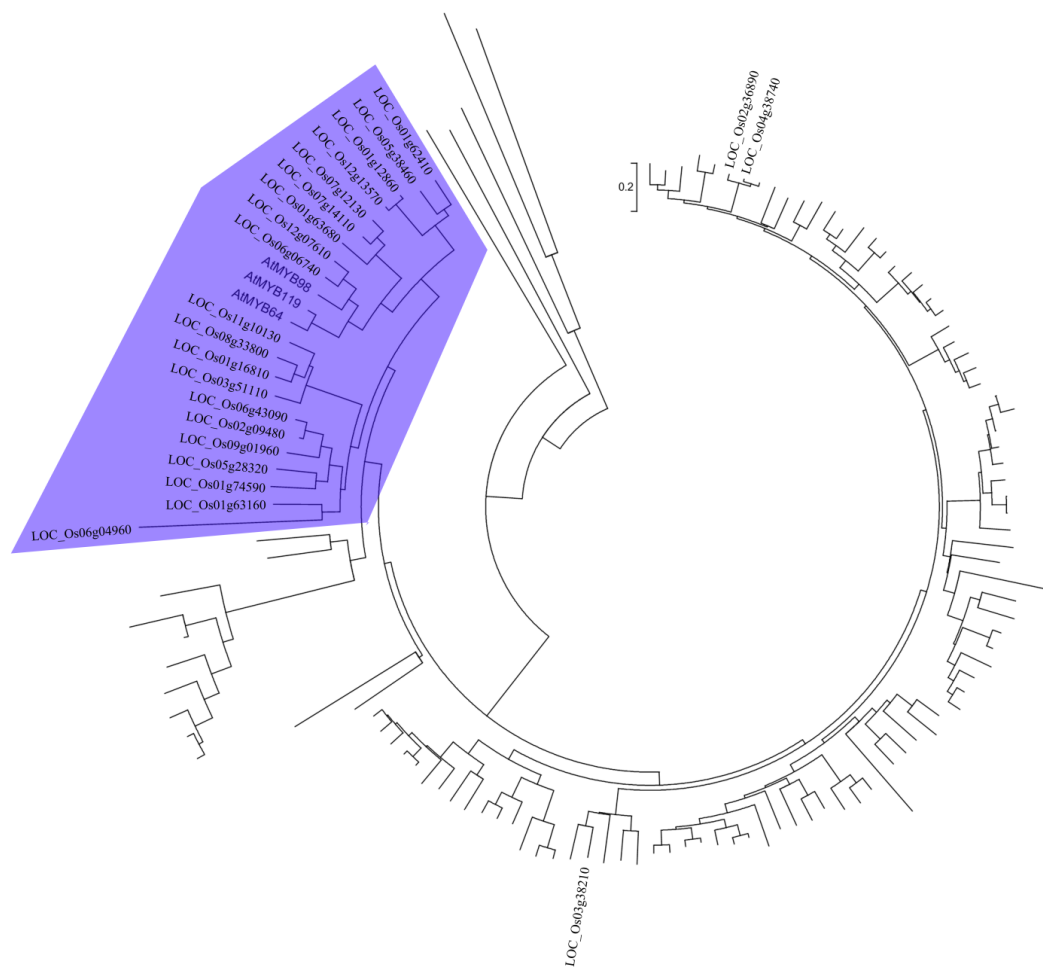

**Supplemental Figure 5.** A phylogenetic tree of rice genes homologous to *AtMYB64/98/119* in the blue region.

Supplement: Additional file 19: Figure S5. — A phylogenetic tree of rice genes homologous to AtMYB64/98/119 in the purple region. (PDF 355 kb) [file 12870_2017_1004_MOESM19_ESM.pdf]
