# Supplementary material for: Identification and analysis of the stigma and embryo sac-preferential/specific genes in rice pistils
Source: BMC Plant Biol. 2017 Mar 7;17:60. doi: 10.1186/s12870-017-1004-8 (PMC5341191; doi:10.1186/s12870-017-1004-8)
Supplement: Additional file 20: Figure S6. — In situ hybridization. (PDF 317 kb) [file 12870_2017_1004_MOESM20_ESM.pdf]

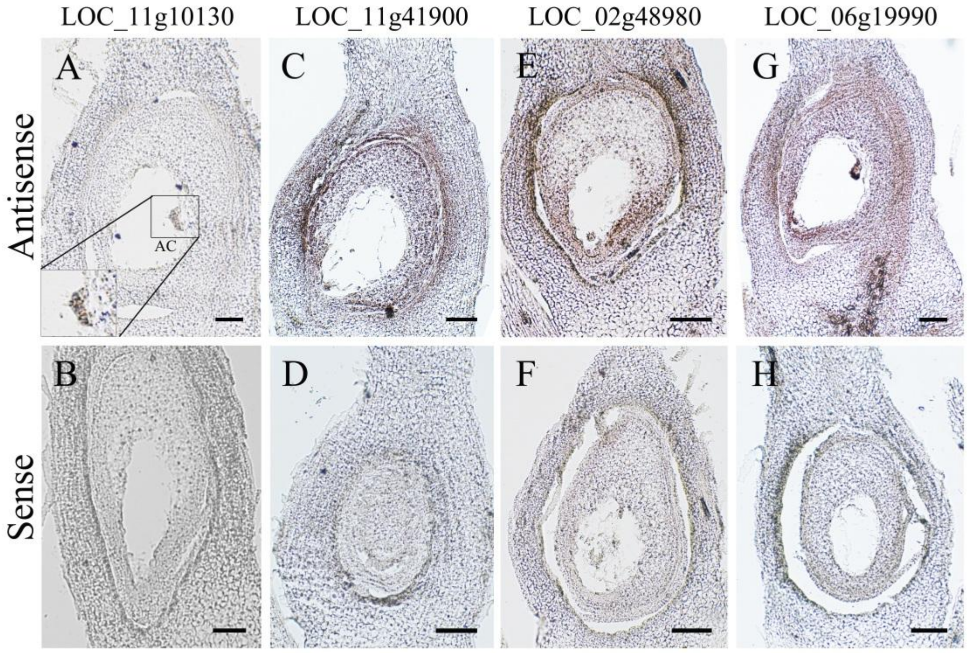

Supplemental Figure 6. *In situ* hybridization. We verified four genes, including LOC_Os11g10130 coding a protein homologous to AtMYB64/98/119 (A-B), LOC_Os11g41900 coding a protein homologous to AtLPAT2 (C-D), LOC_Os02g48980 and LOC_06g19990 coding proteins protein homologous to AtLORELEI (E-H). The results showed that LOC_Os11g10130 was present in antipodal cells but not in the other part of the ovary while the other three genes were present in the entire ovaries, not restricted in the embryo sac. Bar was 100 μm. AC, antipodal cell.
