# Supplementary material for: Identification and analysis of the stigma and embryo sac-preferential/specific genes in rice pistils
Source: BMC Plant Biol. 2017 Mar 7;17:60. doi: 10.1186/s12870-017-1004-8 (PMC5341191; doi:10.1186/s12870-017-1004-8)
Supplement: Additional file 22: Figure S7. — Heat map of rice candidate genes homologous to some Arabidopsis embryo sac-specific functional genes. (PDF 23 kb) [file 12870_2017_1004_MOESM22_ESM.pdf]

Supplemental Figure 6. Heat map of rice candidate genes homologous to some Arabidopsis embryo sac-specific functional genes.

(A) The homologs of *AtFIE*

Gene_ID(RGAP)	Pi-1	Pi-2	*dst* -1	*dst* -2	O-1	O-2	St-1	St-2	At_orth	Reference
LOC_Os08g04290	0.00	0.03	0.00	0.02	0.00	0.05	0.00	0.00	*AtFIE*	Nallamilli et al., 2013
LOC_Os08g04270	59.46	62.26	57.77	51.96	58.54	61.98	54.66	50.96	*AtFIE*	

(B) The homologs of *AtLPAT2*

Gene_ID(RGAP)	Pi-1	Pi-2	*dst* -1	*dst* -2	O-1	O-2	St-1	St-2	At_orth	Reference
LOC_Os11g41900	55.33	52.08	54.08	47.91	60.28	54.91	42.82	43.74	*AtLPAT2*	Kim et al., 2005
LOC_Os01g57360	26.50	25.40	24.50	21.77	26.59	25.20	38.14	38.13	*AtLPAT2*	
LOC_Os05g42270	11.31	8.68	11.04	11.70	11.00	9.33	14.17	14.44	*AtLPAT2*	

(C) The homologs of *AtCCG*

Gene_ID(RGAP)	Pi-1	Pi-2	*dst* -1	*dst* -2	O-1	O-2	St-1	St-2	At_orth	Reference
LOC_Os05g28460	7.79	7.58	8.45	7.77	8.14	8.74	4.94	3.39	*AtCCG*	Kawahara et al., 2013

(D) The homologs of *AtLORELEI*

Gene_ID(RGAP)	Pi-1	Pi-2	*dst* -1	*dst* -2	O-1	O-2	St-1	St-2	At_orth	Reference
LOC_Os02g48980	90.44	83.09	73.71	68.84	76.31	77.21	179.77	212.24	*AtLORELEI*	Kawahara et al., 2013
LOC_Os02g33740	0.00	0.11	0.00	0.00	0.00	0.00	0.13	0.31	*AtLORELEI*	
LOC_Os04g42210	2.38	0.35	1.44	0.00	1.03	0.09	23.06	7.93	*AtLORELEI*	
LOC_Os04g42220	0.11	0.11	0.19	0.00	0.12	0.00	4.10	0.70	*AtLORELEI*	
LOC_Os06g19990	24.66	28.99	41.50	40.81	31.42	21.14	2.60	4.15	*AtLORELEI*	
LOC_Os09g12620	0.30	0.10	1.10	0.00	0.24	0.00	2.53	2.45	*AtLORELEI*	

Supplemental Figure 6. Heat map of rice candidate genes homologous to some Arabidopsis embryo sac-specific functional genes. Numbers in cells represented FPKM values derived from RNA-seq of HY pistils (Pi), *dst* ovaries (*dst*), HY ovaries (O) and HY stigmas (St) in duplicate. At_orth, Orthology in Arabidopsis. All the candidate homologous genes were not down-regulated in the *dst* .
