# Supplementary material for: Identification and analysis of the stigma and embryo sac-preferential/specific genes in rice pistils
Source: BMC Plant Biol. 2017 Mar 7;17:60. doi: 10.1186/s12870-017-1004-8 (PMC5341191; doi:10.1186/s12870-017-1004-8)
Supplement: Additional file 23: Figure S8. — The respective expression levels of stigma-specific genes in HY stigma. (PDF 184 kb) [file 12870_2017_1004_MOESM23_ESM.pdf]

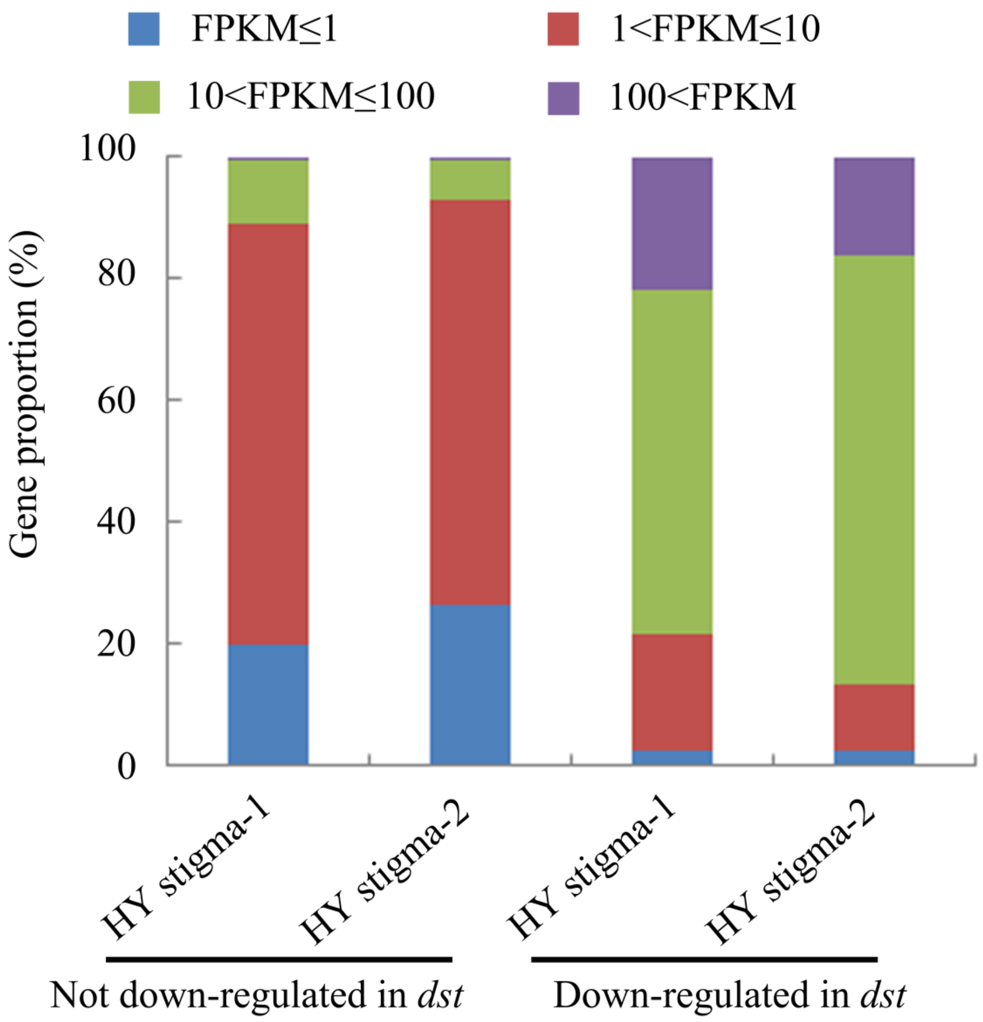

Supplemental Figure 7. The respective expression levels of stigma-specific genes in HY stigma. The expressions of genes down-regulated in the *dst* ovary compared to HY pistil were higher than those which were not down-regulated in the *dst.*
